# Lower limb exoskeleton robot and its cooperative control: A review, trends, and challenges for future research

**DOI:** 10.3389/fnbot.2022.913748

**Published:** 2023-01-12

**Authors:** Gilbert Masengo, Xiaodong Zhang, Runlin Dong, Ahmad B. Alhassan, Khaled Hamza, Emmanuel Mudaheranwa

**Affiliations:** ^1^School of Mechanical Engineering, Xi'an Jiaotong University, Xi'an, China; ^2^Shaanxi Key Laboratory of Intelligent Robot, Xi'an Jiaotong University, Xi'an, China; ^3^Department of Mechanical Engineering, Rwanda Polytechnic/Integrated Polytechnic Regional College (IPRC) Karongi, Kigali, Rwanda; ^4^Department of Engineering, Cardiff University, Cardiff, United Kingdom

**Keywords:** human-robot, cooperative control, exoskeleton, physiological signals, actuator

## Abstract

Effective control of an exoskeleton robot (ER) using a human-robot interface is crucial for assessing the robot's movements and the force they produce to generate efficient control signals. Interestingly, certain surveys were done to show off cutting-edge exoskeleton robots. The review papers that were previously published have not thoroughly examined the control strategy, which is a crucial component of automating exoskeleton systems. As a result, this review focuses on examining the most recent developments and problems associated with exoskeleton control systems, particularly during the last few years (2017–2022). In addition, the trends and challenges of cooperative control, particularly multi-information fusion, are discussed.

## 1. Introduction

Nowadays, ERs are becoming increasingly popular due to improved robotic technologies and the positive perception of people toward interacting with robots. Man-machine collaboration technology can enhance the exoskeleton's convenience or comfort, as demonstrated in rehabilitation studies (Campeau-Lecours et al., 2018; Wu and Li, 2019). Moreover, ERs can be widely used in rehabilitation (Zhang X. et al., 2017), providing power assistance and helping patients in resuming a normal life (He et al., 2018; Yeem et al., 2018; Benabid et al., 2019; Kim et al., 2019; Park and Park, 2019; Xie et al., 2020). For instance, the wearable robot (WR) can play the same role and function as human joints. Typically, it could be used to help people by being aware of the user's intention to perform different tasks. Furthermore, using a multi-sensor network, ERs can collect a patient's movement intention and perfectly cooperate with the user's motion. Also, it can provide external force or torque to the user's legs under control, consequently yielding user-initiated movability (Chen et al., 2017a,b, 2018). The inability to engage in the voluntary movement has likely had several detrimental effects on people's ability to improve their lifestyles in the past and still does so today. For instance, there is a huge limitation to the human's self-dependence, sensorimotor ability, neuroplasticity, and nature of life (Gassert and Dietz, 2018; Wagner et al., 2018; Wang et al., 2019; Bourbeau et al., 2020; Marquez-Chin and Popovic, 2020). In the United States (US) alone, nearly 610,000 people have a stroke for the first time every year, and 185,000 are periodically affected (Salmela et al., 2017). It is estimated that 7.0 million people in the United States under the age of 20 have experienced a stroke, while the prevalence rate for this condition is believed to be 2.5% (Benjamin et al., 2019). Thus, an imperative rehabilitation approach is required to reduce the frequency of deaths. This is because stroke is the leading cause of death and serious long-term disability worldwide (Fernandes et al., 2018). Typically, many people suffer from lower or upper limb paralysis.

Lower or upper limb paralysis reduces people's self-reliance and quality of life, making it difficult for them to perform daily living activities (DLAs; Kim et al., 2017). Patients who are afflicted with low limb motor dysfunction (LLMD) need assistance to perform DLAs. This is one of the most important objectives of neurorehabilitation. Moreover, different areas such as robotics, computer science, clinical treatment, and neuroscience for developing rehabilitation training techniques should be combined to restore motor deficits due to strokes. Truly, without considering the level of disability, we should put forward a rehabilitation approach to regain normal limb movement (Almaghout et al., 2020; Wang et al., 2020). The ability to accurately and quickly restore patients with LLMD's functional movement capabilities to normal is crucial in rehabilitation technology. Besides, because of the aging population, improving the way of life is significant. People with LLMD have reached a huge number; consequently, it can limit their ability to perform DLAs, such as walking, standing up, sitting down, turning right, turning left, grasping, and so on (Liu et al., 2019). As a result, active and passive rehabilitation training modes are the most effective ways to ensure a quick recovery. Rehabilitation focuses on restoring walking ability, mainly in the elderly (Sapiee et al., 2020).

China has the largest number of stroke patients in the world. Approximately 15 million weak people have LLMDs due to conditions such as cerebral palsy, hemiplegia, and paraplegia. In addition, around 40 million disabled people still exhibit a decline in walking capacity caused by aging. Therefore, human-machine cooperation control (HMCC; Ayas and Altas, 2016) is a crucial consideration advanced by many scholars in the past to date to achieve a promising recovery. Their findings show that accurate recovery of motor impairments in a lower limb needs intensive rehabilitation training with different robot-aided devices to create physical interaction between robots and patients in an effective manner (Jamwal et al., 2016; Jiang et al., 2017). However, HMCC still has to make significant improvements. Mainly, the lower limb rehabilitation exoskeleton robots (LLRERs) should effectively combine the following key points: (i) the perception system; (ii) control and command; (iii) different technologies and provide the characteristics of bionics; (iv) robotics; (v) instructions and control science; (vi) medicine; and (vii) different collaborative multi-fields (Ma et al., 2016). As a result, LLRR makes a significant contribution to the recovery of patients with LLMD (Bai et al., 2019; Liu H. et al., 2020). Furthermore, HMCC (Deng and Li, 2018; Mohanta et al., 2018; Yu et al., 2019; Zhao et al., 2019; Farras et al., 2020) is mainly targeted for improvement in 2030 in order to reach a satisfactory recovery stage. Human-robot cooperation control (HRCC) is an important factor to consider in ensuring performance, safety, robustness, reliability, and comfort during rehabilitation (Shi et al., 2019; Xu et al., 2020). HRCC mainly comprises two linked parts: humans and robots. In general, it provides physical contact or interaction between humans and robots (Li et al., 2019a; Zhao et al., 2019). Humans and robots can exchange information about the detected signal and mechanical system through the perception system to enhance the wearer's muscle ability (Liu J. et al., 2017). The main goal is to ensure the performance and comfort of the patient *via* the HRCC system. The objective of the human-robot linkage is for the robot to cooperate with the wearer by quickly and effectively determining the user's intention (Liu H. et al., 2020). Besides, human-robot integration would progressively become the main target for developing a comprehensive smart life. During interaction time, the safety issue would be critical to prevent the robot from creating second-degree damage to the human body (He et al., 2017; Zhang S. et al., 2018). In this regard, the robot's movements need to be carefully controlled.

The robot's varied control mechanisms have been employed for a variety of applications. For instance, they can be applied in industrial, power assistive, and rehabilitation robot areas. The HMCC system can provide movement information control (position, velocity, and acceleration) and produce promising results. However, several methods still show a big gap to be filled that may promote high recognition accuracy. Thus, it is urgent to design robust and effective control algorithms to help identify the user's motion intention and optimally produce the correct trajectories with the WRs. Furthermore, it is possible to improve recognition accuracy by combining two physiological data types, such as EEG and EMG. This study aims to summarize and compare the various HMCC methods based on physiology and conventional cooperative control (CC). In addition, the advantages and disadvantages of each strategy will be discussed.

Physiological signals and data collected through human-robot interaction (HRI) are essential to the success of human movement intention detection methods in an authentic setting. The method is based on physiological data, which means that sEMG (Song et al., 2020a), EEG, or functional Near-Infrared Spectroscopy (fNIRS; Hong et al., 2020) are used to look at how people move. Moreover, these approaches mainly utilize bioelectrical signals-based control due to its promising benefits, such as rapid system response and suitability for detecting active motion intention for lower limb control. At the same time, it can promote outstanding recognition accuracy. Although a few limitations can appear, such as electrode shift, limb position, interference (ambient noise, transducer noise, power line, and so on), muscle fatigue, time-varying contraction intensity, and brain motor damage that cannot generate limb motor control signals. On the other hand, the perfect approach depends on HRI data, namely: capture point (CP), ground reaction forces (GRF), human joint trajectory, interaction force or human joint torque, and plantar pressure or center of pressure (CoP; all these techniques are known as non-bioelectrical signals; Chen et al., 2013; Lajeunesse et al., 2016; Rupal et al., 2016; Parri et al., 2017; Molteni et al., 2018; Zhou et al., 2019; Song et al., 2020a,b; Dalla Gasperina et al., 2021; Huamanchahua et al., 2021; Li et al., 2021; He et al., 2022). Nevertheless, there is a significant disadvantage as well as an inconvenience; in this regard, subsequent motions should be able to achieve the interaction data. Thus, these approaches have a higher chance of yielding a time delay between human movement and interaction data collection. Furthermore, this issue can affect the data processing and robot mechanical system response times. At the same time, it can cause the device-supportive control to lag, which can put the user in uncomfortable situations or promote imperfect motion during rehabilitation. Hence, the physiological approach is better than the traditional one.

The most basic technique is the on-off control, which uses pattern recognition to categorize human intentions into many classes. Thus, the excellent accuracy of human motion pattern recognition (HMPR) employing an LSTM neural network is achieved. Through sEMG signals, it is possible to achieve human voluntary joint torque. However, employing a non-linear model approach is complicated and challenging (Song et al., 2020a). An HRCC approach based on a virtual fixture has been proposed, which adopts an admittance control (AC) algorithm to realize effective HRI between the surgeon and robot during the surgery; the virtual fixture is applied to restrain and guide the robot's movements, thereby ensuring their safety. However, the HRI effectiveness is low, and the training time is prolonged for the active control mode based on voice and head motion, which requires further research (He et al., 2022). Human-robot cooperative control (HRCC) algorithms have been thoroughly examined to bridge the gap between HMC aspects; however, they only focused on the upper LRER (Dalla Gasperina et al., 2021). It has been proposed to use a radial basis function (RBF) neural network compensation control approach based on computed torque. However, it does have a few drawbacks, such as the fact that it can only provide accurate results for non-linear systems, a time-consuming training procedure, and an evaluation of a dynamical model (Yu et al., 2011; Yin et al., 2015). The fuzzy PD position controller has been used to control the whole arm manipulator (WAR). However, this control algorithm is challenging because its fuzzy inference systems are rarely direct (Bai et al., 2017). A perfect HRCC can promote the users' comfort, and guarantee safety, robustness, adaptive performance, reliability, freedom of motion, and optimal control interaction. However, a big challenge is choosing and designing an optimal control algorithm that requires high professional skills. For instance, impedance control (IC) and admittance control (AC) can be used, but more expertise is required to adjust their parameters.

The most important criterion for ensuring HRI is safety. Control systems can be used to enable collaboration between robotics and humans. HRI thus seems to be an appropriate instrument to accomplish safety through constant communication and the interaction of humans and robots. However, the unpredictable contact between the user and the robot, which could promote crashes and dangerous or harmful situations for the user, makes the HRI application a problematic endeavor. In light of this, PD controller algorithms are used because of their simplicity, lack of system knowledge required, and ability to simulate unique human movement patterns by adjusting parameter values. P- and D-terms are applied in the forward path, despite having a large time delay that necessitates parameter modifications; step references provide quick changes and spikes in the control signal, and control signals yield problems or failures with the actuator unit. Several control approaches have been investigated to enhance linkage performance when applying admittance/impedance models, including the impedance adaptation technique based on the cost function and impedance learning based on machine learning algorithms. Nonetheless, these techniques are difficult in improving robot compliance and motion effortlessly while performing pHRI in an unknown dynamic linkage environment. Besides, a few surveys of the literature examined pHRI execution quantitatively.

A trajectory deformation algorithm (TDA) has been investigated, and the HRCC method with a low-level position monitor and high-level trajectory tracking has been investigated for LLRR to produce the required trajectory (Zhou et al., 2021). Truly, the crucial challenge or drawback of the control strategies based on position trajectory or tracking control is that they push the user's limbs on fixed reference or predefined trajectories without considering the user's impairment stage or level with personal adjustments, which is tough to achieve by a therapist or patient (Gilbert et al., 2016; Masengo et al., 2020). Thus, certain automatic adaptation principles can be applied to address this challenge. The critical root of the automatic reference trajectory adaptation algorithms is to change the robot's movement as the user requires. Accordingly, the robot movement is obtained from the mechanical linkage between the user and the robot and computed by the robot force sensors. In order to improve the patient's degree of rehabilitation recovery, the patient's awareness of voluntary movement is highly required, and cooperative control between the rehabilitation robot and the patient is indispensable (Jiang et al., 2019). Moreover, details of typical control algorithms and their challenges are comprehensively discussed in Sections 3, 6, and 7.

Lower limb exoskeleton robot cooperative control (LLERCC) is the main focus of what has been reviewed in this survey. It has been found that the last in-depth survey of the LLERCC was published in 2013 (Chen et al., 2013). Nevertheless, brief studies have been presented in Lajeunesse et al. (2016), Rupal et al. (2016), Molteni et al. (2018), Shi et al. (2019), Zhou et al. (2019), Gull et al. (2020), Huamanchahua et al. (2021), Li et al. (2021), and Rodríguez-Fernández et al. (2021). Besides, the different researchers conducted surveys to highlight the state-of-the-art in ERs (Rehmat et al., 2018; Baud et al., 2021). However, the previously published review papers failed to comprehensively analyze the control approaches, which is a critical aspect of the automation of exoskeleton systems. Thus, this review focused on exploring the latest trends and challenges of exoskeleton control systems, particularly from 2017 to 2022.

This paper is planned as follows: Section 2 presents the introduction and development of the exoskeleton robot and its actuation techniques. In Section 3, a review of traditional cooperative control methods is presented, while in Section 4, EMG-based cooperative control is discussed. In Section 5, EMG and EEG-based control are discussed, whereas Section 6, discusses the trends of cooperative control (multiple information fusion). In addition, Section 7, details the challenges of human-robot cooperative control, while the concluding part of the paper is presented in Section 8.

## 2. Lower limb exoskeleton robot (LLER)

### 2.1. Development of exoskeleton robot technology

The development of ERs technologies has rapidly increased in recent years; however, most of them are still in their infancy. At present, the control of lower limb robots is a popular topic of research in the United States of America (USA), Europe, China, Japan, and Singapore. The United States, Japan, and Europe are now leading in this field, as these regions already have some fairly mature commercial products on the market (Jiang et al., 2017; Pérez-San Lázaro et al., 2020). Furthermore, LLRR devices have been developed to help LLMD patients (Huang et al., 2016). At the same time, significant progress has been made in control strategies, especially in promoting patients to be actively involved in rehabilitation training (D'Agostino et al., 2017; Burkow et al., 2018).

The current control strategies can be divided into on-demand assistance mode, resistance mode, imitating daily tasks mode, and non-contact teaching mode (Chen et al., 2019). These categories reflect the different ways that patients can participate in rehabilitation. The most commonly used technique is the non-contact coaching mode, which is an on-demand assistance method that incorporates the active force of the patient into the control system rather than simply viewing it as LLRR interference (Young and Ferris, 2016). Different training approaches have been proposed in the past. However, many scholars have focused on the exoskeleton control (EC) aspect due to its superior contribution in the military, industrial, and medical sectors. In the medical sector, rehabilitation for long-suffering LLMD patients makes it possible to boost their lower-limb abilities. Robotic bodysuits help people who suffer from mobility issues to enhance their physical movements and restore their quality of life and freedom. The aim of the exoskeleton suits is for the disabled to get rid of those items that create psychological issues. Moreover, WRs are designed to intensify the user's strength and lifespan.

**Figure 1:**
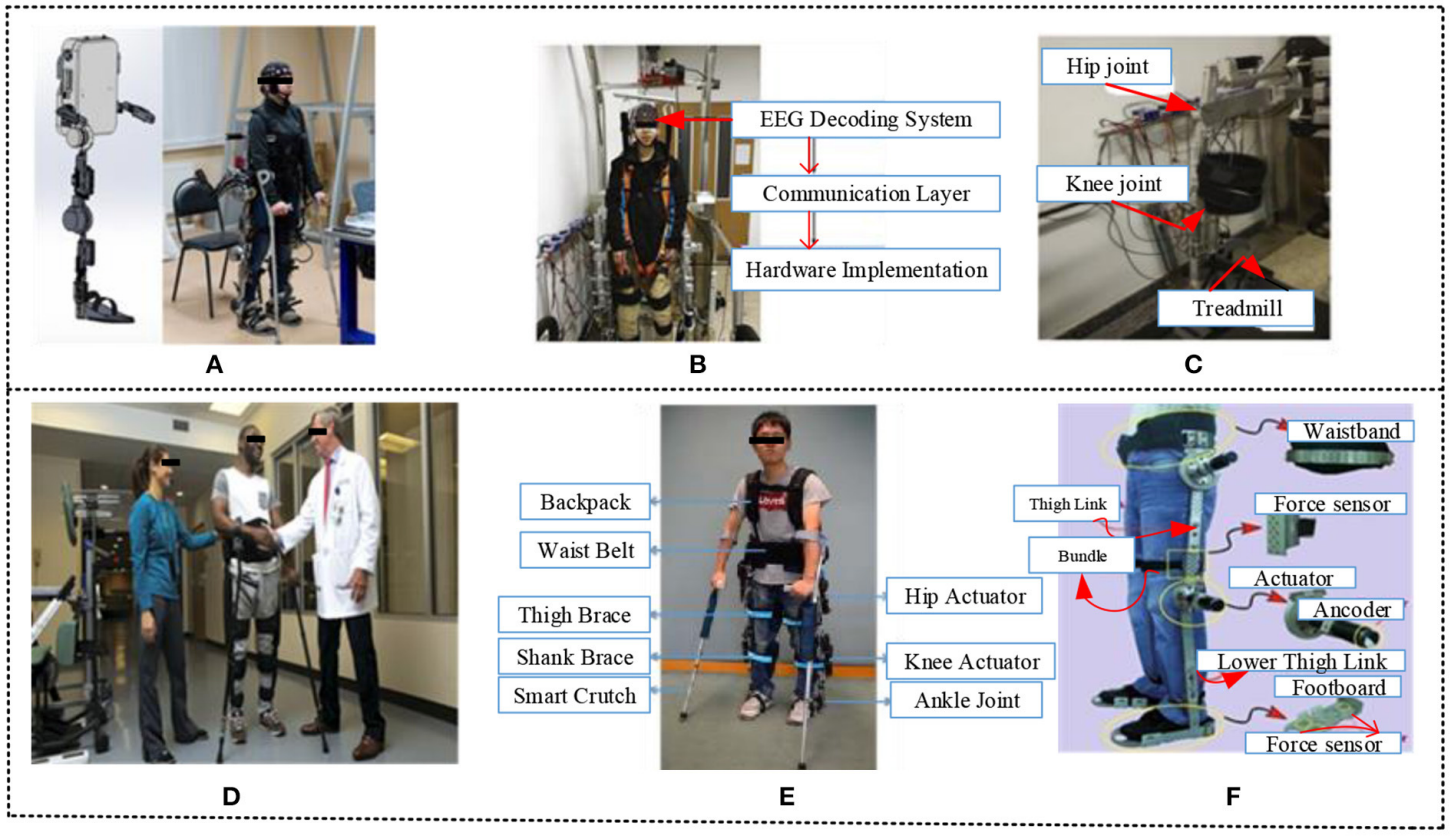
**(A)** Laboratory setup of the lower-limb exoskeleton integrated with mHMI. The monitoring system commands the drives to carry out the needed motions while examining the measurements from the sensors to control the effectiveness of the set motions. The movement can be achieved by controlling information from the EMG and EEG data classifiers (Gordleeva et al., 2020). **(B, C)** Lower limb exoskeleton by the School of Automation Science and Electrical Engineering, Beihang University, Beijing. An HRI loop was introduced, where the information features of the user were decoded and regulated to enhance BCI outcomes. At the same time, position feedback was incorporated into the mechatronics structure (Liu D. et al., 2017). **(D)** Lower extremity exoskeleton by the Human Motion and Control Business Unit of Parker Hannifin, with clinical partners the Shepherd Center, Kessler Foundation, Rehabilitation Institute of Chicago (RIC), and Craig Hospital. LEEs allow patients with disordered movements to regain the ability to perform various motions, such as standing, walking across the floor, and going upstairs and downstairs (Chen, 2017). **(E)** Overview of the Chinese University of Hong Kong (CUHK-EXO) CUHK-EXO, The controller scheme of the human-exoskeleton (HE) system is mainly comprised of high-level controllers and low-level controllers. The high-level controller is used to perceive the wearer's motion intention, to examine and evaluate their motion plan, and then to produce the hip and knee joints' reference trajectories based on the motion conditions. The low-level controller is responsible for operating the actuators based on the produced reference trajectories (Chen et al., 2018). **(F)** Lower extremity exoskeleton robot of the Chinese Academy of Science: The robot adopts an anthropomorphic structural design concept to meet various users' differing wear requirements (Wang C. et al., 2018).

In fact, the use of exoskeletons is currently gaining increasing recognition in the civilian sector, particularly for patients suffering from lower- or upper-limb motor dysfunction. Nonetheless, monitoring exoskeletons usually necessitates a physical method, such as a push button, unlike how regular motor actions are triggered (Wang L. et al., 2018). Brain-wave activities are a significant and promising approach to combining rehabilitation devices with neuroprosthetic equipment. It can be achieved *via* brain-machine interface (BMI) methods that come from EEG signals produced autonomously through external stimulation (Fleury et al., 2020; Ren et al., 2020). Thus, as can be seen in Figure 1A, the LLER has been developed by the Scientific and Production Company MADIN (Nizhny Novgorod, Russia). Exoskeletons have been developed for two goals: restoration and ambulatory support. Three main parts make up the exoskeleton: the mechanical body, control, and sensor structures (Gordleeva et al., 2020). The LLRR was designed at the School of Automation Science and Electrical Engineering, Beihang University, Beijing, as shown in Figures 1B, C, using the brain-controlled method. The structure mainly consists of three essential divisions: the EEG decoding approach, the connection layer, and hardware design, with two closed loops in the system structure.

The paradigm of the biomechatronics structure is shown in Figure 1C (Liu D. et al., 2017). Referring to Figure 1D, using a multi-sensor approach, the exoskeleton can collect the wearer's motion intention while at the same time cooperating with the user's motion correctly. It is possible to provide external force or torque to the wearer's limbs under control and then encourage the user to initiate movement (Chen, 2017). Furthermore, this system can improve the power of users' leg joints. The University of Hong Kong CUHK-EXO has introduced an approach to letting paraplegic patients resume a normal life, as shown in Figure 1E. Based on data collection from paralyzed patients, the most common preference of these people is to regain their capacity to move, particularly their ability to stand and walk. The CUHK-EXO design is mostly composed of essential parts, namely, an exoskeleton mechanical system, actuators, a backpack with an embedded controller and batteries, multiple sensors, and a pair of smart crutches. It weighs around 18 kg and can be used continuously for about 3 h (Chen et al., 2018). The Hefei Institute of Intelligent Machinery, Chinese Academy of Sciences, has introduced the lower extremity exoskeleton (LEE) robot design, as shown in Figure 1F. This robot device platform consists of four main parts: a power unit, LEE, an actuation system, and a multi-sensory perception system. Each leg of the ER has three joints, the hip, knee, and ankle, with 12 degrees of freedom (Wang C. et al., 2018).

**Figure 2:**
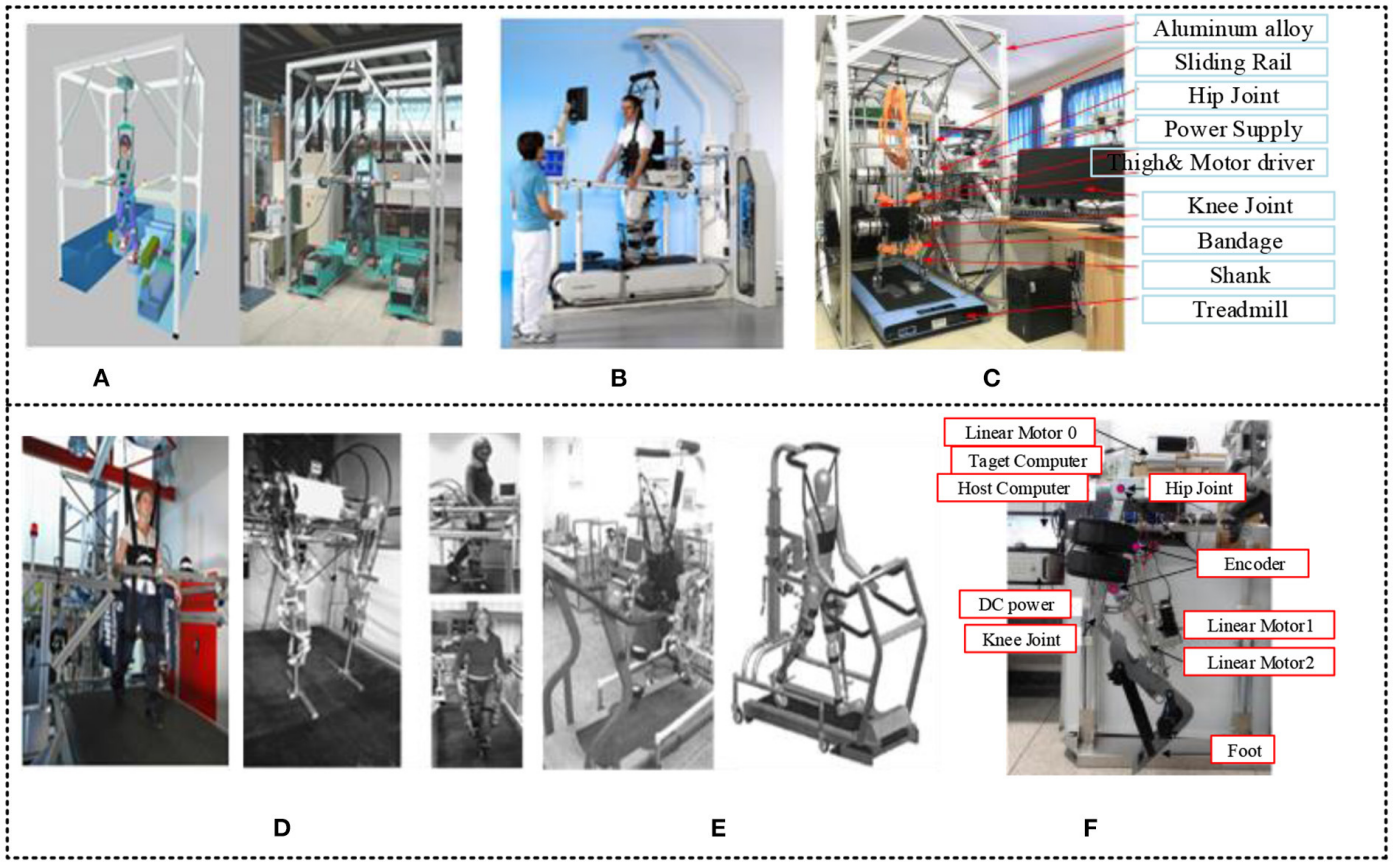
**(A)** A novel haptic device by Fraunhofer Institute IPK, Berlin, Germany, Department of Automation and Robotics, the Haptic Walker. This haptic locomotion interface is skilled at reproducing slow and smooth trajectories, simulating walking on the floor and up and down the stairs. It is a footplate-based training device and possesses the training mode of passive, active, and resist. Position control and impedance control are used (Schmidt et al., 2005; Chen et al., 2014). **(B)** Lokomat. The Lokomat's hip and knee joints are initiated by linear drives incorporated in the exoskeletal design. A passive foot lifter prompts ankle dorsiflexion during the swing phase (Fang et al., 2020; Lin et al., 2020). **(C)** Lower extremity rehabilitation exoskeleton platform. The exoskeleton is composed of two mechanical lower limbs (Chen et al., 2020). **(D)** LOPES or treadmill-based LLREs. The LOPES has superior performance due to the innovative design of the joint drive, which uses a flexible cable drive instead of the traditional drive (De Rossi et al., 2010). **(E)** The LLRE robot developed by Zhejiang University. A semi-automatic control strategy can be based on the patient's gait during training for exclusion and adaptation of gait curves with appropriate amendments to reduce patient discomfort in the rehabilitation process (Lyu et al., 2016; Pinheiro et al., 2020). **(F)** The mechanical framework of the exoskeleton (Lyu et al., 2016). Briefly, many robotic rehabilitation techniques consist of robotic external skeletons adapted for a particular body part connected to the program, which sends information and data training to the exoskeleton and vice versa. The amount of assistance or force contributed by the robot can be adapted, and the systems come with pre-programmed routines that can be set to the patient's level of movability.

A novel haptic device for walking simulation with a locomotion interface was designed at Fraunhofer Institute IPK, Berlin, Germany, Department of Automation and Robotics, as shown in Figure 2A. Two programmable foot platforms with regular foot-machine communication make up the framework. Locomotion haptic interactions can permit strides and other foot motions inside virtual environments (Schmidt et al., 2005; Chen et al., 2014). The Lokomat was designed by the Rehabilitation Institute of Chicago in the 2000's and is used to train patients with a damaged central nervous system (CNS) in therapeutic walking (Fang et al., 2020). The Lokomat is a bilateral robotic orthosis combined with a body-weight support system to manage the patient's leg motions in the sagittal plane, as shown in Figure 2B (Lin et al., 2020). The developed lower extremity rehabilitation exoskeleton framework in Figure 2C depends on bionics methods. The exoskeleton is connected on two rails to the aluminum alloy skeleton outside. The exoskeleton is composed of two mechanical lower limbs (Chen et al., 2020). On the other hand, the New Zealand Biomedical Engineering Laboratory at the University of Auckland designed the gait rehabilitation robot LOPES (lower extremity powered exoskeleton); all design criteria have been considered, as shown in Figure 2D. The device, known as LOPES, combines a freely translatable and 2-D-actuated pelvis segment with a leg exoskeleton containing three actuated rotational joints: two at the hip and one at the knee. It is lightweight and actuated by Bowden cable-driven series elastic actuators (De Rossi et al., 2010). An LLRER and gait training simulation schematic diagram has been designed by Zhejiang University, as shown in Figure 2E (Pinheiro et al., 2020). The system structure consists of buffering, a backrest, weight suspension, treadmills, and LEE composition. The lower force sensors implanted in Lokomat can be used to obtain torques at the knee and hip joints. The mechanical framework of the lower limb exoskeleton was designed as shown in Figure 2F. The system platform consists of linear motors, a host computer, a target computer, and a DC power supply. Furthermore, the exoskeleton has been attached to three absolute encoders for feedback data acquisition. One is attached to the hip joint, and the two others are attached to the upper ends of the linear actuators (Lyu et al., 2016).

### 2.2. Exoskeleton classifications and actuation approaches

**Figure 3:**
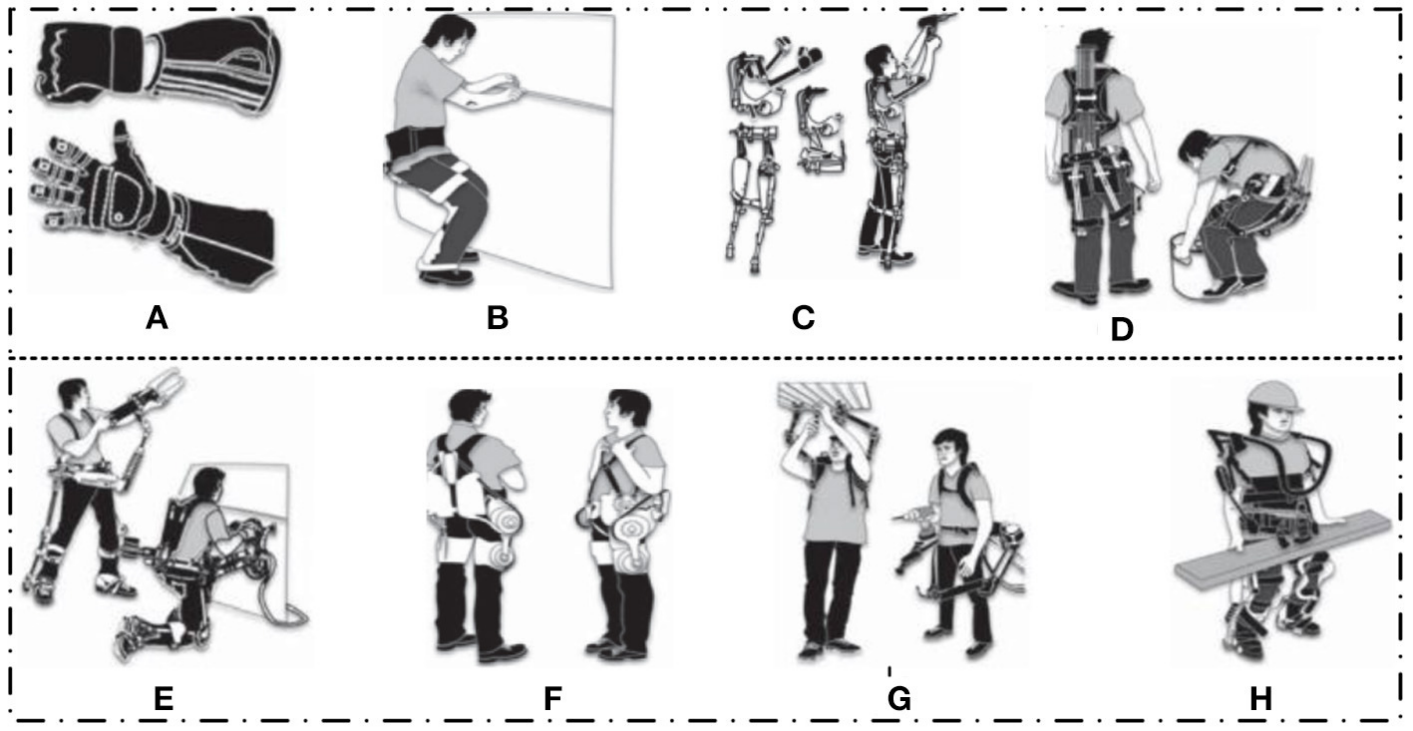
Exoskeletons with multiple application purposes (Fox et al., 2019). **(A)** Exoskeleton gloves (Yun et al., 2017) can minimize the operator's force to grasp a tool and provide assistance during the execution of a task (Yurkewich et al., 2019). **(B)** Chairless chairs, which are wearable chairs consisting of two supports for the backs of the legs that touch the ground when wearers bend their knees to sit. **(C)** Exoskeletons for the shoulders (Islam et al., 2020), back, and legs can be manufactured. This can decrease human musculoskeletal pressure from repetitive, light overhead work. **(D)** This exoskeleton structure can contribute to and yield supporting power through the use of carbon fiber rods, which act as artificial tendons by bending when the wearer squats and springing back when they stand up. **(E)** Exoskeletons can deliver direct help for heavy hand tools by offloading their weight onto external support, such as a floor, *via* a series of linkages at hips, knees, and ankles, bypassing the wearer's body. **(F)** A powered exoskeleton's ability to communicate or combine different materials, such as batteries, sensors, actuators, and motors, is crucial (Ismail et al., 2019). **(G)** Powered exoskeletons can expand the human body by supplying and influencing energy to the arms and legs. In **(H)** the exoskeleton platform can be designed in an improved manner and made strong to maximize the loads that can be supported (Cho et al., 2018).

**Figure 4:**
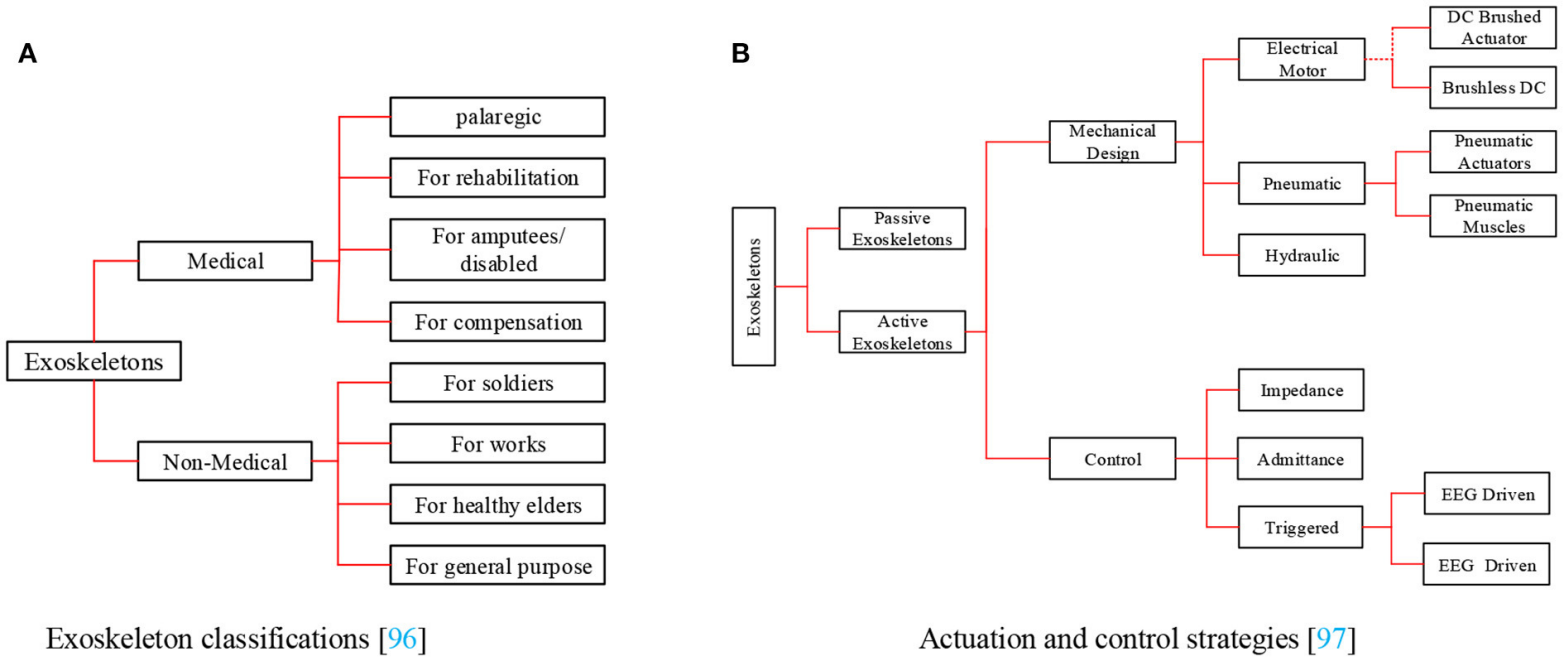
Exoskeleton classifications and actuation approaches. **(A)** Exoskeleton classifications (Rupal et al., 2017). **(B)** Actuation and control strategies (Frisoli, 2018).

As shown in **Figure 4A**, there are two types of ERs: restorative (medical) and non-restorative (non-medical; Del-Ama et al., 2018) approaches. General treatment (medical) exoskeletons are considered to provide or boost joint/limb movement in some particular applications where capability is inadequate or lost in movability and power. Moreover, those exoskeletons embrace ankle exos for drop-foot uses or hip and knee exos for restoration targets (Chang et al., 2016). In contrast, non-restorative robots (non-medical exoskeletons) can physically assist healthy people in performing different tasks, as shown in Figure 3 (Fox et al., 2019). For example, it can boost laborers' ability to perform physically demanding duties, like the military's ability to carry heavy loads and to achieve a high speed over rough terrain (Nussbaum et al., 2019). Furthermore, those ERs can help regular people perform daily chores, such as assisting older people with DLAs and encouraging them to maintain an active and productive lifestyle.

There are various technical aspects to consider when making lower and upper LERs. Thus, the choice of actuation approach is a crucial point to put forward. The distinct technological alternatives for actuation design techniques are detailed in Figure 4B.

Furthermore, an appropriate actuation mode should be capable of satisfying the following requirements: small size, lightweight, low power consumption, and high-power output, and it should have various advantages such as quick response, low inertia, high precision, and excellent safety. Usually, the most common driving methods are SEA drives, motor, hydraulic, and pneumatic actuation (Junior et al., 2016). Firstly, a hydraulic actuation system can provide a suitable power density. However, it can cause some security issues, such as oil leaks due to the enormous pressure needed by the oil system, and it is not always willing to guarantee user satisfaction (Jiang et al., 2017). Secondly, various designs use pneumatic actuation, where power is transmitted using air, and this contributes to clean pollution, fast response speeds, and easy maintenance. However, because air is compressible, this design results in lower transmission accuracy and poor stability velocity, and it is not easy to control (Chiang and Chen, 2017; Huang et al., 2020; Zhao and Song, 2020). It should be taken into account that the motor drive mode can promote various advantages such as easy control, high movement precision, fast response, low cost, ease of use, high driving efficiency, no noise pollution, among many others. Besides, electric motors have a number of advantages. For instance, they are usually utilized in brushless DC motors to decrease electromagnetic discharge, which is needed for medical tools. Thus, it is widely used in medical equipment (Masengo et al., 2020). Finally, a SEA (Series Elastic Actuator) drive mode can offer the following advantages: high control precision, high safety, weakened inertia impaction lowering, and friction losses storing energy, but it can also result in some downsides, such as huge power consumption, rigidity limited by elastic elements, wide volume, quite weighty, and complex design (Zhang T. et al., 2018; Li and Bai, 2019). Different types of actuators are presented in Table 1.

**Table 1:** Summary of different types of actuation modes.

**Actuation mode**	**Description**	**Benefits**	**Drawback**	**References**
Pneumatic	Using compressed atmospheric air as the triggering means to generate power and control.	Low-cost, clean, fast air is non-explosive and non-toxic, and compressed air can be stored; a pressure limit switch can be used in a compressed air system to promote easy maintenance.	Air collects moisture, so drying may have to be considered; the speed can simply vary under the charge, and the exhaust air causes noise. It may be necessary to use sound-absorbing materials because highly steady, smooth movement is not impractical and is unsuitable for a high-power system.	Chiang and Chen, 2017; Huang et al., 2020; Zhao and Song, 2020
Hydraulic	The use of pressurized fluid as triggering means activating power transmission and monitoring.	More genuine, capable of preventing rust and corrosion, lubricating moving parts, dissipating heat, and removing unwanted and harmful impurities from the system, more stable, promoting promising inertia, with a secure overcharge, and easy to control.	High oil leakage; low drive speed; high pollution; low viscosity due to high temperature and low energy efficiency.	Jiang et al., 2017
Servo Motor	Utilizing electric equipment and adjusting network values for energy transfer and command.	Simple control, excellent movement precision, quick response, minimum price, easy to apply, promising driving outcomes, no noise pollution, simple structure, and other various benefits.	It shows a low stability impact from outside charge, more inertia, and weightiness.	Masengo et al., 2020
SEA drive	In a series of elastic actuators, an elastic element is attached to the mechanical energy source output.	High control force accuracy, high security, reduced inertia impaction, shock tolerance (lowering abrasion removal), energy conservation, low output impedance, increased peak power output and ensuring the stability of the human-machine linkage.	Rigidity is limited by elastic tools, huge volume, hefty weight, complex design, huge power, and significant complications in vibration suppression.	Zhang T. et al., 2018; Li and Bai, 2019

## 3. Traditional cooperative control

Robots are typically controlled in a programmable manner by detecting information and using artificial intelligence approaches (Perez et al., 2018). An algorithm should control these kinds of robots to predict the state of the environment. Nevertheless, such control methods have limits on robot purpose and performance. Many scholars have advocated developing exoskeleton robot structures, which are human force enhancement technologies (Peng et al., 2017). This exoskeleton enables users to physically use the robot's forces. Furthermore, an exoskeleton robot model is useful in different industries, such as medicine, rehabilitation, and the military. For instance, soldiers can use them to minimize or remove the strain (Hamza et al., 2020) on their backs. Through the exoskeleton platform, users can handle the robot's posture and movement recognition and generate execution signals (Liu G. et al., 2020). Thus, an exoskeleton structure for force assistance behaves similarly to the user's joints (Sun et al., 2019). The HRCC of an ER should typically be aware of the control information system for the robot's motions and the force produced through those same motions. During the coupling of humans and robots, the linkage movements can create an interaction force. Additionally, the patient's effort should be considered by enabling the machine to act in accordance with forces impacted by the patient itself. It might help the robot achieve flexibility and adaptation. The interaction is improved *via* a multimodal display and a virtual reality-produced environment such as haptic, visual, and audio (De la Iglesias et al., 2020). Therefore, HRI can facilitate rehabilitation, which is beneficial to patients for a speedy recovery.

During exoskeleton control (Young and Ferris, 2016), the user makes significant contributions in the area of HRCC (Kardan and Akbarzadeh, 2017), such as task command generation, environmental feeling, and feedback control of robot movement information. Thus, the ER motion controller should recognize the required user movements and execute them accordingly. However, traditionally, the motion control issue for an exoskeleton robot can be detailed by computing the joint torque and the required position. As a result, to control such a mechanical scheme, the inverse dynamics (Arora and Bera, 2019) control is used, which is targeted at linearizing and decoupling the robot dynamics through a feedback signal (Abu-Dakka and Saveriano, 2010). Furthermore, non-linearities like Coriolis, centrifugal, and gravity torques can be approximately eliminated by adding these factors to the control input. On the other hand, decoupling can be done by giving the inertia matrix more weight than the control input.

**Figure 5:**
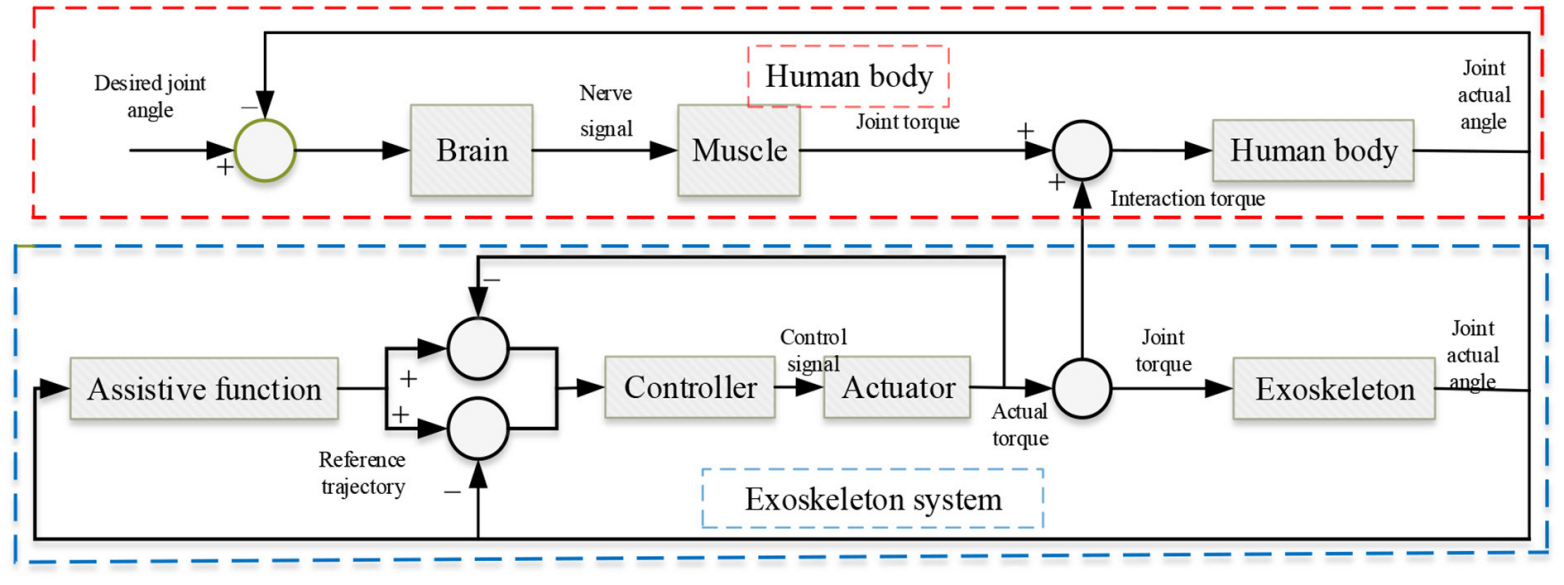
Structure of conventional human-exoskeleton cooperation (HEC). In the exoskeleton mode section, conventional torque or standard linkage trajectories can be reached based on a determined or predefined supportive function. The desired input torque is connected with the controller to generate control information to drive actuators. The resulting electromagnetic force of the actuator should be utilized or employed to push the user and the exoskeleton. Consequently, the coupling force or energy will be applied to the user's linkages as additional support for the reference movement. In addition, when the user provides desired movement information, their brain can yield nerve information based on perceptive feedback of the real movement. Finally, users can motivate their muscles to provide or produce joint information data such as force or torque (Chinmilli et al., 2017).

Generally, integration of typical user CC approaches is highly challenging when the user and robot are conventionally cooperative (Li et al., 2019b). For instance, the well-known conventional approach uses IC laws, allowing the user to deviate from any predetermined reference trajectory with a variable virtual spring-damper element (Arora and Bera, 2020). In most applications, the goal is to allow the robot to recognize the subject's intended movement and permit it as a solution instead of imposing a preset motion. Based on Figure 5, the exoskeleton is connected to the user and incorporates a human knowledge or perception system and mechanical force capability. Furthermore, the use and device result in a closed-loop system with a CC approach. Typically, in the control techniques of exoskeletons for the gait recovery process, two key methods are necessary: trajectory control (or tracking) and assistance. For instance, in a trajectory tracking control method, the user's preset trajectories of the leg joints can be used for control. However, this control method has more limitations because the users are passively trained without considering their motivation, which can actually promote faulty rehabilitation (Chinmilli et al., 2017).

Different CC methods have been proposed by previous researchers. In Li K. et al. (2020), a hybrid impedance controller (HIC) based on generalized momentum has been proposed for tracking dynamic contact force and recording non-contact axis position. Moreover, tremendous force tracking features and high position flexibility in the position control line can be optimally achieved. In Duan et al. (2018), a novel adaptive variable impedance control (AVIC) has been investigated for dynamic contact force detection in an uncertain environment. Besides, this approach can provide huge benefits, such as unknown environmental stiffness and dynamic environmental location evaluation. It can also be applied to slope surfaces or other complex areas, and it yields a hugely convincing solution to the contact operation. In Zeng et al. (2019), the active compliant control method has been used to confirm that robot assembly is an effective method. Furthermore, PD-based position control as the inner control loop and IC as the outer control loop have been investigated. The outcome has proven that the algorithm is feasible and can provide outstanding flexibility and positional accuracy. In Yu et al. (2011), Yin et al. (2015), and Tao et al. (2016), a radial basis function (RBF) neural network compensation control approach based on computed torque has been proposed. The main target has been to achieve trajectory tracking with outstanding precision. The uncertainty of computed torque and the disturbance of rehabilitation can be eliminated based on RBF techniques, and the tracking ability is well-enhanced for rehabilitation training. Beyond that, RBF is easy to fabricate, has outstanding generalization, a vigorous tolerance to input noise, and a simple topology; it can also provide good online learning ability.

As described in Van Tran et al. (2015a,b), the indirect drive gait training rehabilitation robot Walkbot is made of two main parts: harmonic drive transmissions and a timing belt. Compensation methods and the reaction torque observer (ROB) have proved crucial for estimating patient-robot interaction torque. Furthermore, the proposed technique can satisfy and meet the requirements of patient-cooperative control, in which the system can detect patients' voluntary efforts and allows them to be actively involved in gait patterns during rehabilitation exercises. Finally, the control system was not difficult to design, and its accuracy is excellent.

In Chen et al. (2020), a patient-cooperative rehabilitation training approach based on adaptive impedance monitoring has been crucial for the swing phase of the training. Human–exoskeleton interaction torques have been checked by a BP neural network and a disturbance observer whose balance has been confirmed by Lyapunov's law. A fuzzy algorithm has been utilized to motivate the patients to be involved in rehabilitation exercises as the key point to modifying the impedance parameters based on human–exoskeleton interaction torques (HEIT). In addition, the effectiveness of the proposed control strategy can guarantee that those patients who have suffered from a stroke will actively participate in rehabilitation training.

In Zhang et al. (2019a), the consequences of a mental fatigue state on the execution of brain-controlled prostheses have been examined. It has been determined that the effectiveness of the classification is significantly reduced by the subject's mental fragility. Consequently, it can decrease the performance of the brain-controlled prosthesis, which is dependent on the facial-expression model. Moreover, the researcher has found that it's crucial to set up algorithms that can adapt to the variation in the subjects' cognitive behaviors and enhance the strength and feasibility of BCIs. EEG contingent BCIs, event-related synchronization and desynchronization (ERS/ERD), mu and beta rhythms, P300 visual evoked potentials, and steady-state visual evoked potentials (SSVEP; Xie et al., 2017) can all be considered rehabilitation techniques. Thus, in Zhang L. et al. (2017) and Zhang X. et al. (2017), a novel object-oriented SSVEP-BCI model has been tested. This system promotes the employment of a continuous action scenario involving the monitored object to change conventional input to stimulate the outcome of SSVEP, which enhances SSVEP's precise recognition and maximizes a user-friendly BCI system of “what you detect is what you obtain.” In (Zhang et al., 2019b,c), an eye-tracking approach based on the asynchronous SSVEP-BCI technique has been used to promote the BCI system procedure by direct localization and asynchronous eye-tracking-based switching to expected stimulation intent. Moreover, by integrating the mixed signals of eye gaze position with a traditional asynchronous BCI system through the conducted method, the system can provide the following benefits: minimal trial duration and significantly improved identification accuracy. In Rotier et al. (2018) and Aljalal et al. (2020), the study produced a portable brain-machine interface device that is suitable for brain-controlled wheelchairs and also an HMC system with an acceptable interface. The control system mainly comprises the acquisition system, which collects the user's brain signals, the corresponding software for signal refinement and analysis, and the hardware side for receiving and transmitting control command signals after deciding. The system is also simple, effective, accurate, and applicable. In Lv and Gregg (2017) and Lv et al. (2018), a perfect hypothesis structure for the under-actuated energy shaping technique—which changes the mechanical system's characteristics and integrates environmental and human interaction—has been conducted to offer human-cooperative exoskeletal assistance. The typical trajectory-based control has provided the user with the least amount of voluntary control over the device, limiting its usability for different patients. Thus, the proposed approach can overcome this issue by producing task-invariant, trajectory-free control laws suitable for different DLAs. In Bai et al. (2017), the HRC technique has proven to be crucial; the fuzzy PD position controller has been used to operate the whole arm manipulator (WAR) to execute rehabilitation training steadily and smoothly. Here, differential regulations can successfully maximize the dynamic of the system's performance, while proportional regulations can reduce the divergence. However, there are still concerns that need to be addressed, such as the difficulty of precise modeling and the lack of stability proof. Table 2 presents different conventional cooperative controls and their comparisons.

**Table 2:** Comparison of various conventional cooperative control.

**Control algorithms**	**Advantages**	**Challenges**	**References**
1. HIC	Tremendous force tracking features and high position flexibility.	Improper adjustments can create system instability.	Li K. et al., 2020
2. Navel AVIC	Dynamic contact force detection in an uncertain environment, unknown environmental stiffness, and dynamic environmental location evaluation can also be applied to slope surfaces or other complex areas. It can yield a hugely convincing solution to the contact operation.	Complex.	Duan et al., 2018
3. PD-based position control	Feasible, and it can provide outstanding flexibility and positional accuracy.	It only works well for linear systems, and a large time delay process is required for parameter adjustments.	Zeng et al., 2019
4. Radial basis function (RBF) neural network compensation control	The uncertainty part of computed torque, as well as the disturbance of rehabilitation, can be eliminated, which will optimize tracking ability.	Only the nonlinear system can be effectively trained without a time-consuming training process and dynamical model evaluation.	Yu et al., 2011; Yin et al., 2015; Tao et al., 2016
5. Compensation methods and reaction torque observer (ROB)	The technique can satisfy and meet the requirements of patient-cooperative control, it is easy to design, and has good accuracy.	-	Van Tran et al., 2015a,b
6. Adaptive impedance monitoring with BP neural network	The patient can effectively participate in rehabilitation training.	-	Chen et al., 2020
7. Under actuated energy shaping technique	The approach can overcome this issue and yield task-invariant, trajectory-free control laws that can be suitable for the various DLAs.	Complex.	Lv and Gregg, 2017; Lv et al., 2018
8. Fuzzy PD position controller	It is stable and smooth, minimizes deviation, and optimizes the system's dynamic performance.	Rarely are fuzzy inference systems used directly in the control loop and sometimes low control.	Bai et al., 2017
9. Trajectory deformation algorithm (TDA)	TDA can promote the trajectory of rehabilitation training more naturally and smoother, enhance robot compliance, and protect the user from secondary injury.	The tracking trajectory's smoothness is significantly worse than that of the desired trajectory.	Zhou et al., 2021
10. Fuzzy NN	Excellent at handling uncertain information.	It cannot handle the vague the message, slow processing speed, low accuracy, and massive linear and angular velocities that lead to oscillation problems.	Bai et al., 2017

## 4. Human-robot cooperative control (HRCC) based on physiological signals

Depending on whether or not the user's voluntary intention is taken into consideration during the rehabilitation process, exercises for rehabilitation may fall into one of two categories: passive training or active training (Fereydooni et al., 2020). It has been investigated and proved that the active training approach, which promotes the patient's voluntary intention to be involved during rehabilitation training, is a highly effective and successful technique for neurorehabilitation and motor recovery (Maier et al., 2019). Thus, to set up active training, the user's motion intention needs to be identified, which can be achieved by employing physiological signals, including surface electromyography (sEMG; Ma et al., 2020) and electroencephalogram (EEG) signals (Jeong et al., 2020). Both signals can provide unprecedented levels of control over an individual's self-reliance. However, in monitoring assistive equipment, the main challenges are still at the surveillance and cooperation stages. The biggest question that has yet to be resolved or considered is how to design strong and effective algorithms that can optimally determine the patient's motion intention recognition and produce accurate trajectories with wearable robots (Jimenez-Fabian and Verlinden, 2012). Table 3 presents a summary of EEG&EMG recognition accuracy after various fusion approaches.

**Table 3:** Summary of EEG and EMG recognition accuracy after various fusion approaches.

**References**	**Subjects**	**EMG (accuracy)**	**EEG (accuracy)**	**EEG + EMG (accuracy)**	**Fusion approach**
Lóopez-Larraz et al. (2018)	20 patients	65.8 ± 14.3%	59.6 ± 7.5%	69.5 ± 11.0%	-
Shusharina et al. (2016)	10 healthy men	74.3%	-	86.8%	-
Bogdanov et al. (2016)	10 healthy	74.3%	-	86.8%	-
Leeb et al. (2010)	-	83%	77%	91%	Bayesian fusion
Sargood et al. (2019)	-	61.0%	57.78%	69.95%	Believe theory
Tortora et al. (2020)	11 healthy	75%	-	< 80%	-
Leeb et al. (2011)	12 healthy	87%	73%	91%	Simple fusion

The use of conventional rehabilitation approaches (Liao et al., 2020) can easily result in low accuracy due to their poor effectiveness in the interaction between the exoskeleton system and the patient. In most conventional rehabilitation systems, the actuation and processing techniques used in a master-slave relationship (Aliff et al., 2012) tend to force the patient to respect only predefined standard trajectories without considering personal characteristics, spontaneous intentions or voluntarily made attempts. For example, most actuated orthoses communicate with the patient's legs to follow a predetermined movement pattern but do not respond to the patient's voluntary attempts. In addition, in the conventional approach, the patient remains passive, and their intentions are disregarded rather than taking into perfect consideration their complete sensorimotor system (Gilbert et al., 2016). To overcome this issue, a new rehabilitation technology has been introduced in Riener and Munih (2010). The authors presented a novel technique for incorporating humans into a closed-loop system in which the user is highly involved by giving commands to a device. Furthermore, humans control the system by providing biomechanical (Benoussaad et al., 2020) and physiological feedback.

**Figure 6:**
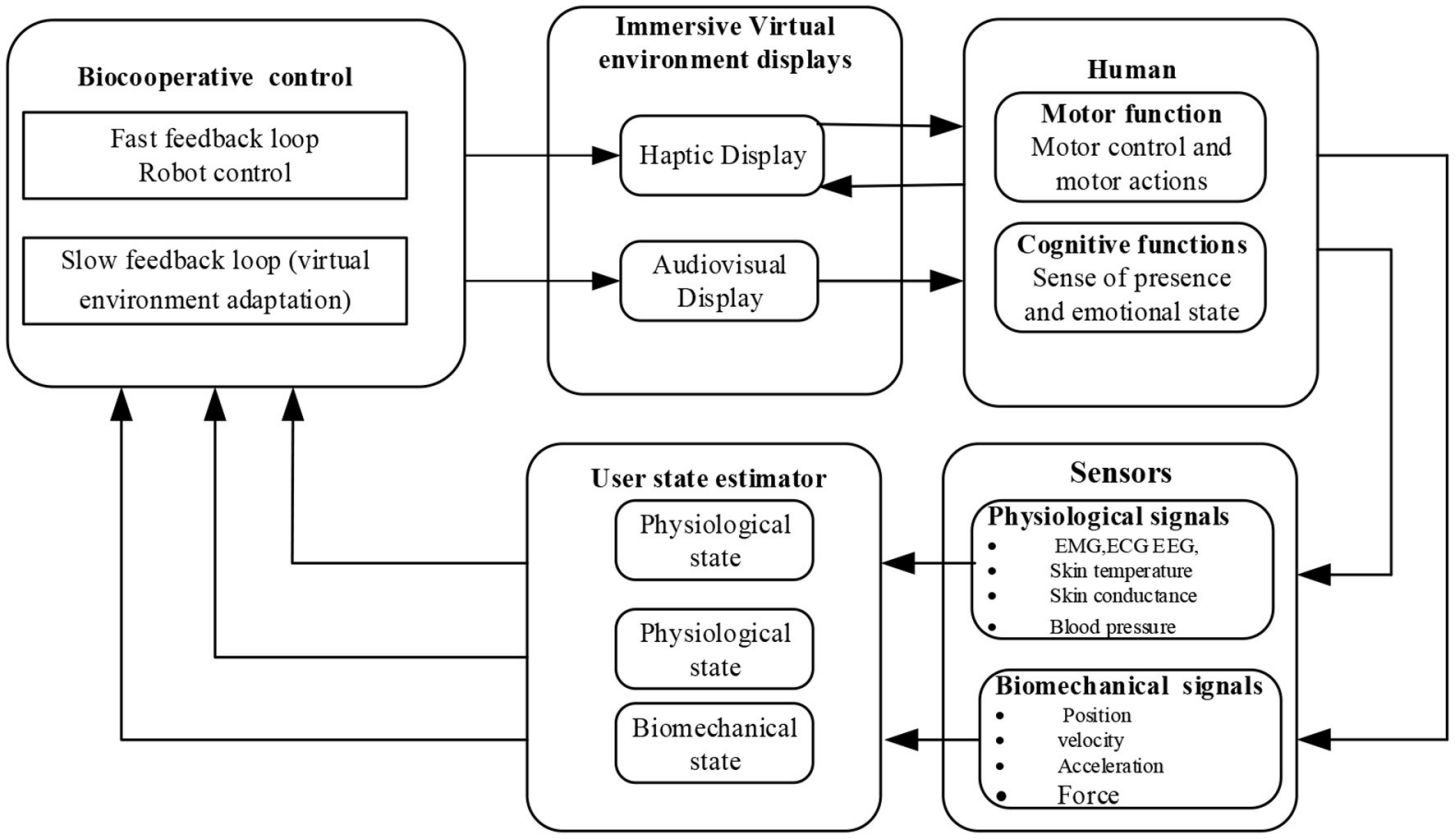
A human-robot bio-cooperative control system. Humans are in the loop regarding biomechanical, physiological, and psychological aspects (Riener and Munih, 2010).

The interaction transformed into a bi-directional and practical rehabilitation mode when the user's characteristics, intentions, actions, and environmental factors were considered. Again, the audiovisual display of a training system should be tailored to the patient's awareness to optimize involvement and increase motivation, as shown in Figure 6. So far, to integrate the user into the loop system, three main points have been considered, including biomechanical, physiological, and even psychological. Biomechanical integration improves the rehabilitation system to be more secure, comfortable, and user-friendly (Bingjing et al., 2019). As a result, rehabilitation robotics should be considered: the robot compliantly assists the human, helps the patient in achieving guaranteed safe interaction, and adjusts the force of the interaction as needed, to allow the patient to contribute to the motion with their voluntary attempts (Tu et al., 2020; see Figure 6).

**Figure 7:**
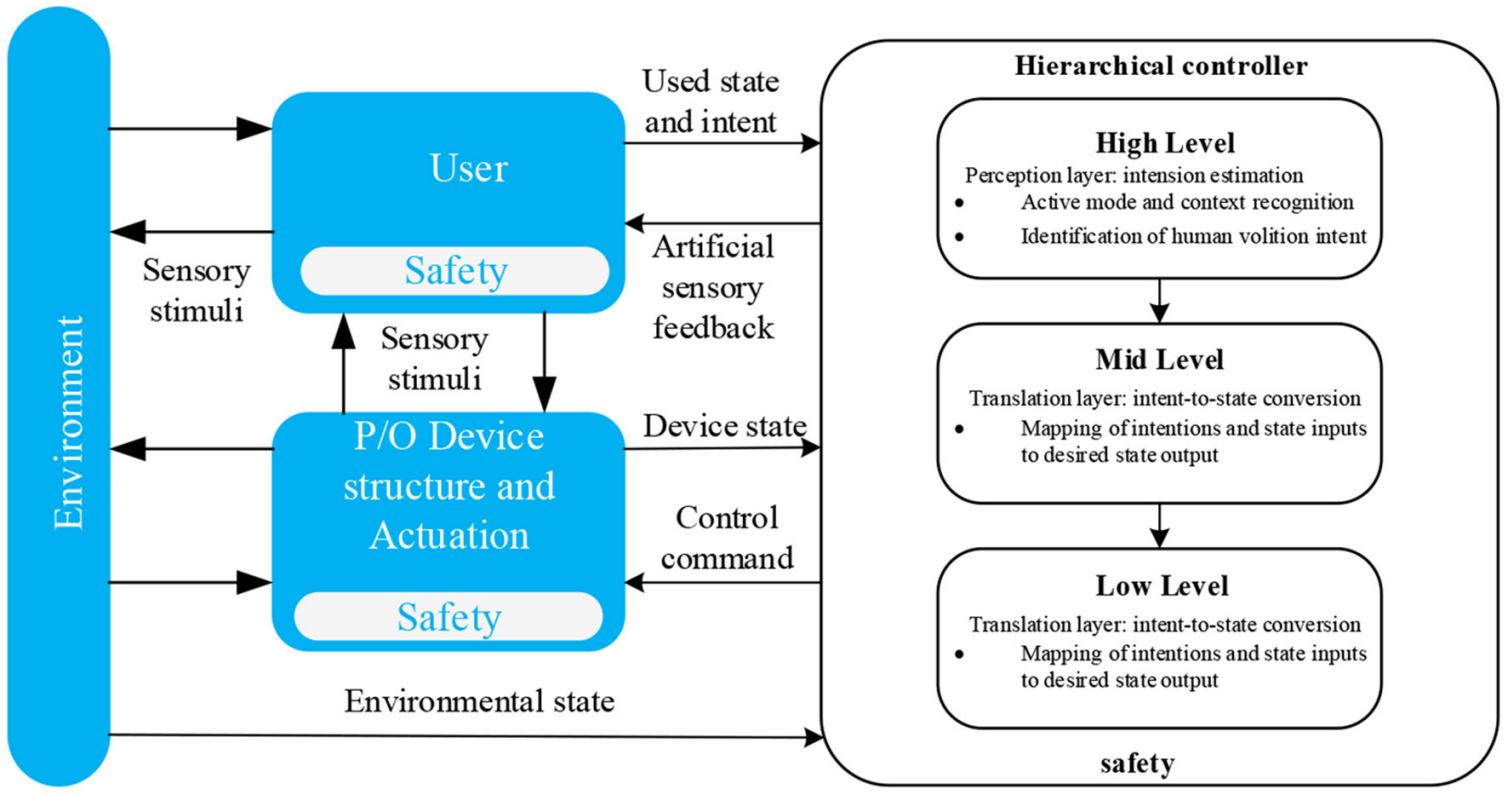
Generalized control framework for lower limbs. Three stages are essential for LLEC: high-level, mid-level, and low-level. First, the layer accomplishes action mode recognition on the high-level stage, enabling the controller to switch between mid-level controllers suitable for various locomotive duties, such as level walking, stair ascent, standing, hopping, and so on. Second, at the mid-level stage, a layer converts human intentions into instructions or estimate points that are then transferred to local controllers, each placed at every linkage of the exoskeleton. Finally, in the low-level stage, the layer accomplishes real-time control in each joint by performing feed-forward and feed-backward control loops (Tucker et al., 2015).

The control techniques used in monitoring exoskeletons are typically employed to determine the users' intentions. A summary of HRCC to be considered is presented in Table 4. The majority of control techniques employed in the exoskeleton's control area must consider user-device interaction signals to determine the patient's intention. At a high level, the system can deal with the sEMG information collected from the user and estimate the movement class using a human motion intention perception or recognition system (see Figure 7). With a finite state machine (FSM), the output signals from the HMIR are used to choose a state, defining both the desired admittance and the parameters for the velocity and trajectory of low-level controllers to power the knee joint of the exoskeleton (Vantilt et al., 2019).

**Table 4:** Summary of HRCC to be considered.

**Control strategies**	**Definition**	**Benefits**	**Challenges**	**References**
EMG based control	EMG signals are described as electrical activity generated by skeletal muscles. By considering measurement techniques, sEMG is commonly divided into sEMG and iEMG. sEMG is the signal acquired *via* an electrode attached to the skin area, and iEMG is the signal collected through a needle electrode inside the muscle tissue under the human skin.	sEMG is easier to obtain, has no complex mechanical structure design, can react to the action of a specific muscle group, can monitor and control limb movement more precisely, and the coupling control through sEMG data has greater flexibility, sensing, and resolution than operative force information, inspiring it to detect active movements intent for the lower limb.	Easily affected by interference such as ambient noise, transducer noise, power lines, and so on, so it requires the use of a filter and a high-precision measurement system. The recorded EMG signals are time-varying, and it is generally recommended to synthesize the tasks of multiple muscles to achieve the active movement intentions of subjects.	Zhang et al., 2012; Li et al., 2018; Mohebbi, 2020
EEG based control	An EEG wave signal is the potential activity from the brain achieved *via* electrodes attached to the scalp, which shows the voltage fluctuation generated through ion flow that mediates the neurons within the brain.	The EEG-based interactive control is equivalent to reconstructing the transmission path of brain control information outside the body, using motor and FES equipment as actuators to regain the user's control of limb motor skills, and it can provide high recognition accuracy. It is not limited by the severity of limb disabilities.	This approach is not feasible for paralyzed people with abnormal brain motor functions. In other words, brain motor damage cannot generate limb motor control signals. Beyond that, the changes in expression, emotion, and awareness can surely affect the EEG information produced by the brain.	Jochumsen et al., 2019; Su et al., 2019; Tryon et al., 2019
Impudence based control	IC algorithm is defined as a way of ensuring compliance between the Human-Robot Interaction (HRI) control systems.	It can be employed as a safe interaction target, adaptively for safe interaction motion, and to increase joint control flexibility in effective control systems with advanced physiological actuators. An individual can handle muscle stiffness to cooperate with the interaction forces, and it can help regulate the force between the robot and the environment.	Wrong adjustment of impedance filter parameters can yield unstable contact and generate hard pressure on the target environment when it is not well-adjusted or fixed, and the interaction forces are indirectly controlled by selecting the desired impedance dynamics, which require accurate knowledge of environment parameters, and the issue of time-varying parameters will promote system instability.	Khoshdel et al., 2018; Jalaeian et al., 2020
fNIRS-based BCI systems	fNIRS is a non-invasive approach for measuring hemodynamic information from the human brain or measuring human brain activities.	This approach can offer several benefits, such as having little effect on noise, being portable, light, low cost, safe to employ, easy to wear, not being invasive, having a high temporal resolution, and being a reasonable solution to real-time imaging.	This technique can meet some challenges, such as choosing the right brain activity for patients and a suitable brain region that can promote incorrect rehabilitation.	Hosseini et al., 2018; Asgher et al., 2020; Hong et al., 2020
Admittance based control	Mechanical admittance control (AC) is the inverse of mechanical impedance; it can be defined as the ratio of the input velocity (motion) to the output force.	Effective for force tracking and able to provide significant assistance as needed in the robotic rehabilitation field.	It has time-varying behavior with external disturbances, and calibration errors will lead to trajectory deviation.	Jiang et al., 2019

**Figure 8:**
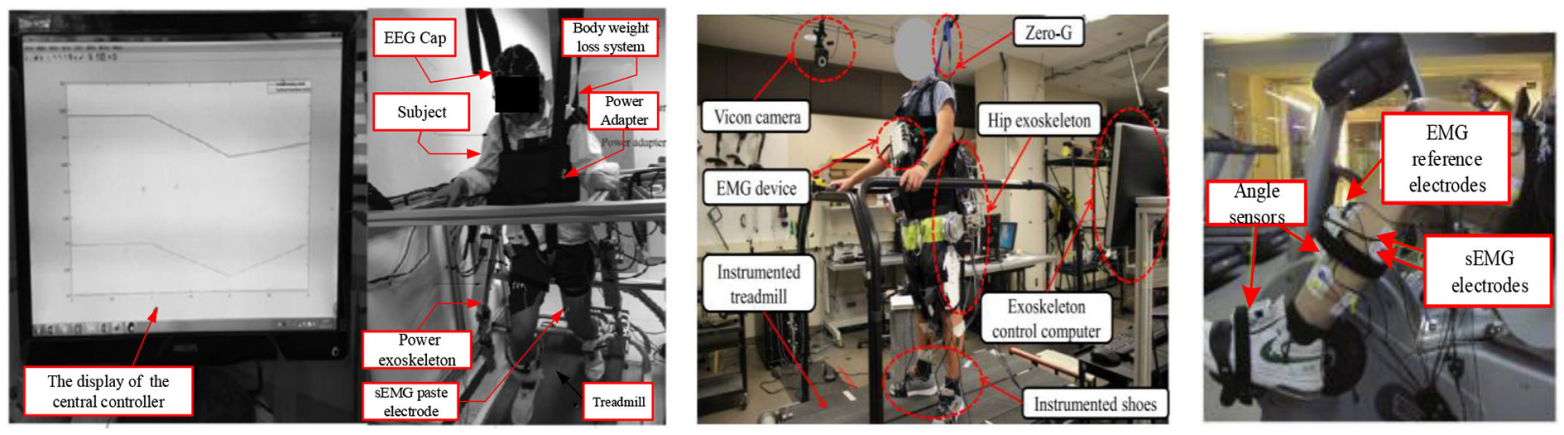
**(Left)** The RT experiment, in which voluntary or active motor training is more effective than passive motor training in eliciting enhancements and cortical reconstruction (Gui et al., 2020). **(Middle)** Lab facilities for a human subject experimental protocol (Zhang et al., 2019d). **(Right)** EMG signals and joint angles acquisition during treadmill training from a non-disabled user (Zhang et al., 2012).

In Yin et al. (2017), an adaptive UKF-based parameter evaluation of a compliant man-machine dynamic model for an LLRR has been investigated. The man-machine-linked dynamics model has been realized based on the relationship between the patient and the restoration robot interface to imitate the user's lower limb movement or to obey the natural conditions of human body movement. The results showed that the parameters of the man-machine dynamic model, which responds to human motion compliance, are estimated online by the adaptive UKF algorithm and provided (Rothe et al., 2020) a significant accuracy. In Gui et al. (2020), a CC approach has proved to be the best technique to enhance the effectiveness of robot-assisted training and motivate patients to participate in more active, voluntary movement during training. It has been provided in a suitable and guaranteed manner, as shown in Figure 8. In Zhang et al. (2019d), the assistive control of the exoskeleton must allow leg movement with the user's intention and voluntary efforts for individuals that retain a certain level of motor control as shown in Figure 8. Due to an active rehabilitation approach based on patients' intentions (Li et al., 2018), a new intention-based bilateral training system using physiological signals representing muscle activity data and active movement intention has been conducted to boost rehabilitation training (RT) outcomes. In Zhang et al. (2012), the hip, knee, and ankle joints' angles have been estimated using sEMG information features to create an active interface for exercising the contralateral lower limb, as shown in Figure 8.

## 5. EMG and EEG-based control

**Figure 9:**
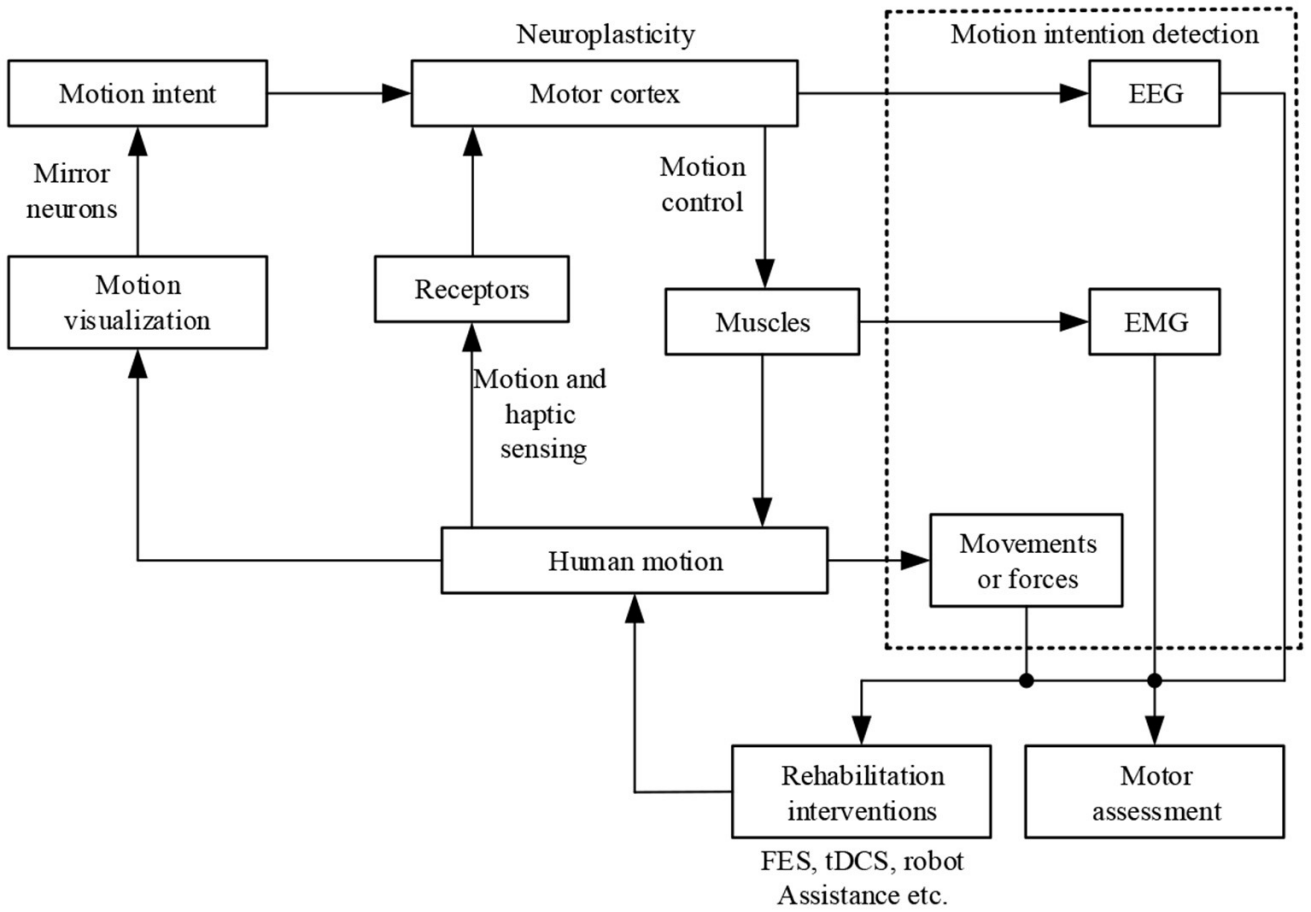
Human intent-controlled motor reconstruction for stroke victims (Li et al., 2018).

Sensors can recognize human movement intent when providing control information between human-machine cooperative behaviors such as motions and forces, as shown in Figure 9 (Bhagat et al., 2014). It is crucial to be aware that the patient actively participates in motor training to generate action- or movement-dependent neuroplasticity. Since EEG signals are independent of residual muscles and contain high-quality neural information on an individual's intentions, developments in BCI systems are designed to control the robot using a subject's thoughts alone (Li H. et al., 2020).

**Figure 10:**
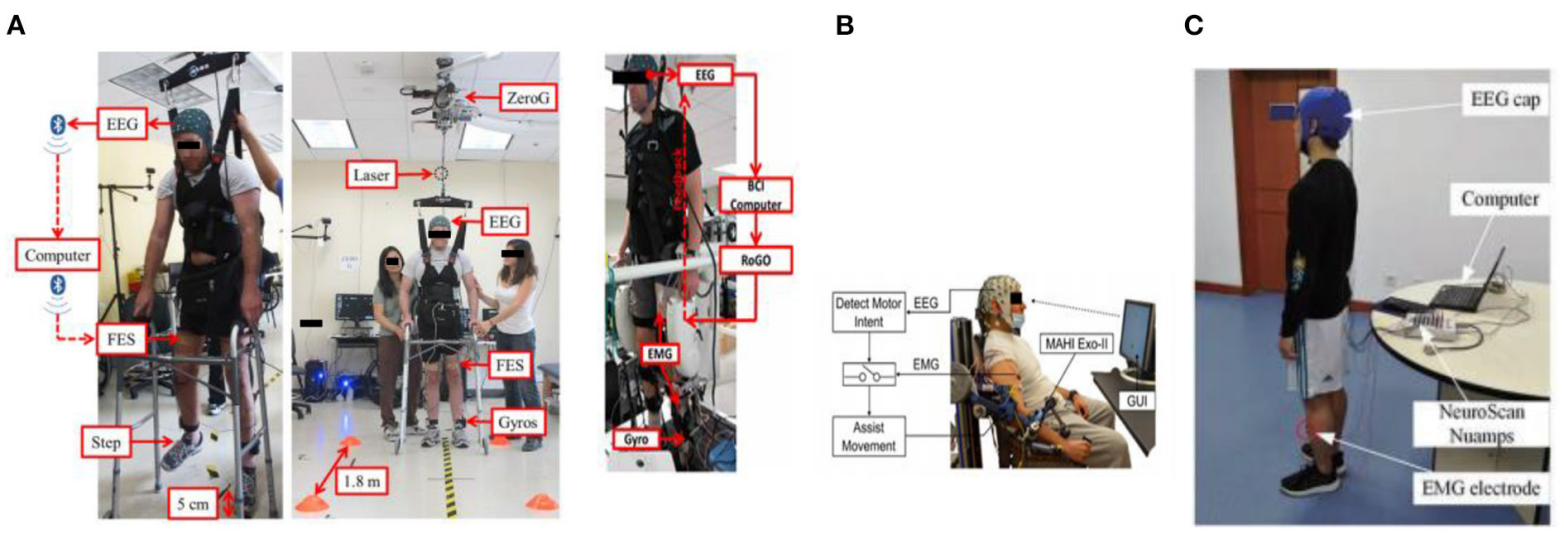
Examples of robot control-based BCI **(A)** EEG-based BCI to control a functional electrical stimulation (FES) system for overground walking (King et al., 2015). **(B)** BCI-robotic gait orthosis (RoGO) and BMI control task (Do et al., 2013). **(C)** EEG electrode position based on the international standard 10–20 system experimental scene (Li H. et al., 2020).

Brain-machine interfaces (BMIs) have been applied to replace or rehabilitate function. Tetraplegic users have employed BMIs to control robotic/prosthetic limbs and FES to carry out tasks and grab or grasp objects. BMIs can perform the restoration function process after a stroke. Hence, stroke patients utilized biofeedback of the mu-beta sensorimotor rhythm to shift the hand using a rehabilitation device (Do et al., 2013; King et al., 2015). Specifically, the goal of most BMIs is to restore function in people with impairments due to neurologic conditions. Moreover, the stability of brain signals is necessary for designing high-promising BMIs, as shown in Figure 10.

**Figure 11:**
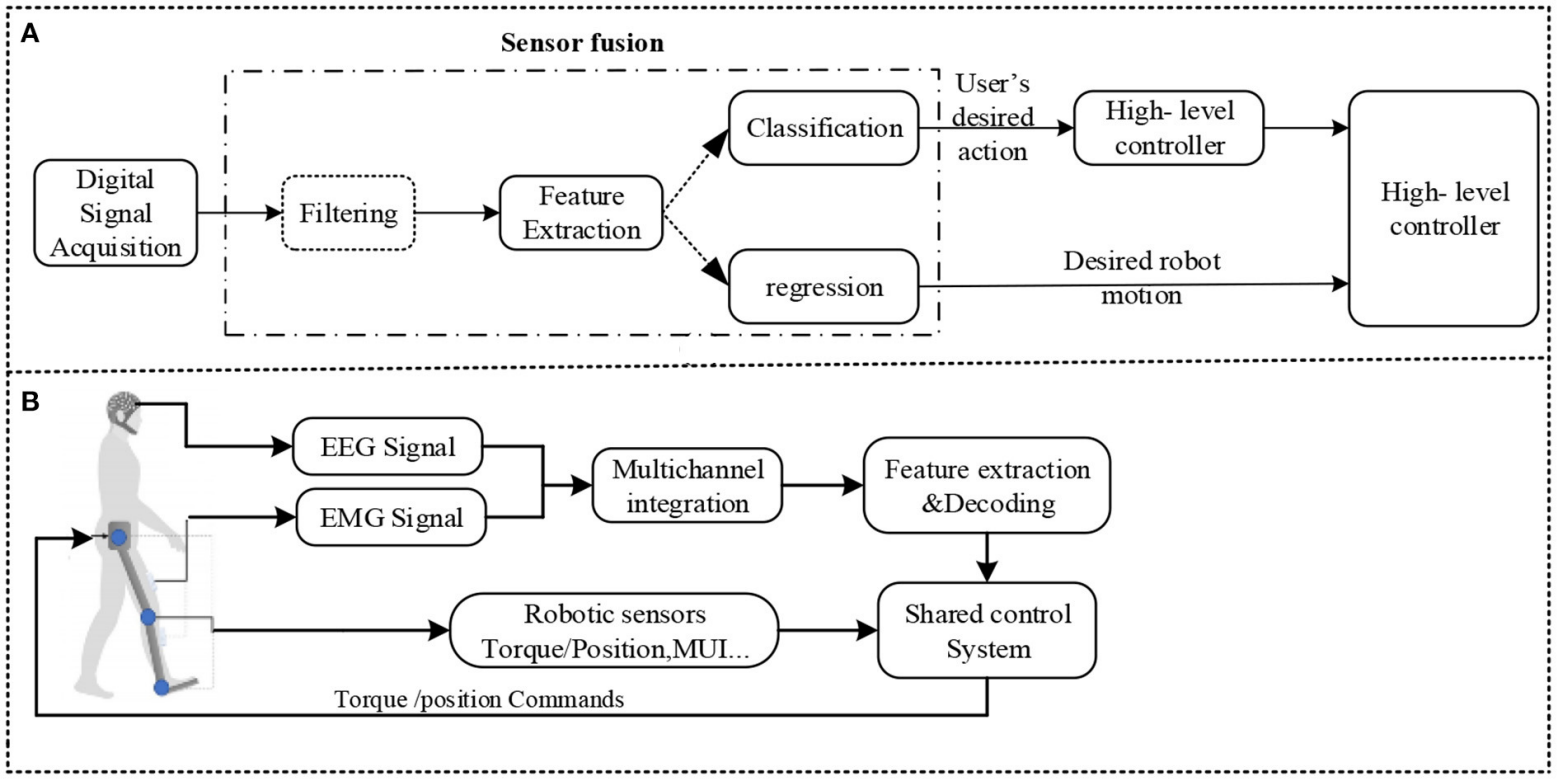
**(B)** EEG-EMG fusion-based cooperative control (Mohebbi, 2020). The following aspects are crucial: filtering, feature extraction, classification, and regression, which are mainly combined to build a sensor fusion approach. Filtering should be performed as the initial step in the sensor fusion process. A wearable device can use classification or regression. Numerous features are simultaneously used as inputs. A high-level device controller is essential to determining how to respond to the user's needed activity. The high-level controller outputs the velocity/torque the robot must use, while **(A)** low-level controller must be aware that the velocity/torque is being employed, as shown in this figure. Regression can transform features into enduring values, such as coupling torque (Su et al., 2019).

The level of control information provided to the end-users by the new wearable robot technology is not comprehensive or intuitive enough. As a result, many scholars believe that sensor fusion algorithms can provide outstanding assistance, make quick response changes to the user's intentions, or improve the accuracy of recognition. In this regard, a multimodal Neural-Machine Interface (NMI) combining EEG and EMG data information to achieve promising control over modern prosthetic legs is required. Fusion techniques can be employed in specific detection modalities [primarily electromyography, electroencephalography, and mechanical sensors (see Figure 11); Mohebbi, 2020].

In Lóopez-Larraz et al. (2018), EEG and EMG fusion has proven to have the following benefits: first, the combined result can ideally guarantee and boost reliability performance while classifying the user's intention. In addition, the hybrid approach can push patients to trigger their complete motor system from the brain to the muscles, traveling *via* the spinal cord and promoting the sensory and motor pathways to function again. An EEG and EMG feature hybridization approach has been investigated to prove the feasibility of combining both bioelectrical data for extending human lifespans (Shusharina et al., 2016). Furthermore, the outcome has proven that the method can strongly achieve a better control signal. In Bogdanov et al. (2016), the combination of EEG and EMG techniques has been highly boosted, reaching an inspiring and reliable outcome. For instance, the outcome was about 12.5% better than when only one strategy has been used. For severe motor dysfunctions, combining EEG and EMG is the way forward. Unequivocally, the classification accuracy has proved to be quite valuable. However, the EEG processing method still requires an advanced hybrid BCI.

The multimodal fusion approach merged brain control with different residual motor features to control information. Thus, the proposed method can successfully achieve valuable accomplishments. For instance, EEG and EMG activities were 77 and 83% accurate, respectively, while after being combined their accuracy reached 91%, a remarkably enhanced outcome (Leeb et al., 2010). Actually, EMG and EEG (Jochumsen et al., 2019) are the two kinds of bioelectricity most frequently employed as biosensing systems in the field of robotic technology. However, it is inadequate to apply a one-modality approach due to enduring artifacts, wrong signals, et cetera. To improve the control signal, this proposal presented two system modalities. The results have shown that the classification accuracy for EMG and EEG was 61.0 and 57.78%, respectively, while improvement reached 10.56% above (Sargood et al., 2019). The Bayesian fusion technique was crucial to combining EEG and EMG features by decoding the walking phases of two lower limbs. The suitable recognition accuracy was above 80% (Leeb et al., 2011; Tortora et al., 2020).

## 6. Human-robot cooperative control trends

One of the most important considerations while monitoring the exoskeleton is the collection of the user's intent features. Generally, EMG and EEG features are the most significant control signals in robotics (Li et al., 2018). These features can be achieved by determining or computing cooperation data between the users and the robots. Decision planning can be achieved through cognitive techniques due to the collected or recognized data *via* several sensory approaches, including auditory, visual, and tactile sensors (Tryon et al., 2019). The exoskeleton robot impacts the cognitive procedure for musculoskeletal structure, while human joints initiate movement.

It is crucial from the LLER's perspective of further control to focus on the challenges associated with the human-exoskeleton coupling system, such as force, torque, power, and data exchange. This can effectively make users feel comfortable and guarantee their safety. Moreover, when well-investigated, they can promote robustness, adaptive performance, reliability, freedom of motion, and optimized control interaction between users and exoskeleton robots. Thus, a good HRI system with an effective control approach must provide an accurate mechanical energy source for the exoskeleton, which should be rooted in or based on kinematics and kinetics data. A low-level controller should determine the physical linkage, and a high-level controller should determine the cognitive transfer. Furthermore, the user–robot coupling of the wearable device may be well-designed and well-controlled as a key element or significant way of quickly and effectively achieving the human's objective. The required control approach diversity is usually determined by the amount of muscle force that the user can produce and the amount of vital force that the supportive robot must supply to the user. In this concept, the power assistive control increases the employed human effort (force) to complete a task, and the wearers are expected to be qualified to exert as much muscle power as possible to initiate the motions. Thus, the controllers must detect the user's force and intensity. These control approaches can be classified as IC, which allows for the modification of the connection force between a human and an assistive robot to provide comfort when the user is being controlled by the robot. On the contrary, in rehabilitation situations, when the patient's capability is not enough to produce adequate force, the controller should produce the force/position pattern. The patient then bases their movements on it, with minimum muscular power.

The user–robot combination adaptation is the essential technique that would be considered for further study. Truly, this is the key to achieving or boosting robot-patient cooperative control. In this regard, robots must be harmonized (integrated) with the patient's prompt movements. In addition to this, the implementation of this concept will stimulate the necessity to identify the movement prompts presented by the patient and suitably adapt the support device and coupling forces. Typically, to cope with human ability restoration, it is crucial to focus on control techniques such as active-assisted control, challenge-based control, haptic simulation, and non-contact coaching. Hence, this would result in a promising stage of vital restoration. A human exoskeleton connection platform often operates in a user loop, which consists of three main parts: the user, the user-robot interconnection, and the exoskeleton. The wearable device is a common HRI structure that is usually in charge of sending information and power to the intermediate user and device. The information or data transfer needs a sensing approach (EEG, EMG interface) to be aware of the user's movements and send it to the device monitoring or control system. The power transfer comes from the device, which can yield the power demanded to support the user's motion based on the user's intent. Thus, future scholars must pay more attention to the user's desired movement estimation technique, which is vital to achieving a suitable device assistance control method. Again, it will become increasingly significant when improving the durability and practicability of recognition techniques, which could be an essential point of future research.

Most of the existing power assist controls are based on impedance control and bioelectrical signals; among them, IC is unsuitable for the support stage. The control-based bioelectrical signal has strong fuzziness, poor anti-interference, and low control precision. Both the IC and the control based on bioelectrical signals divide the gait cycle into the support and swing stages, which are difficult to adapt to different stages of the gait cycle itself. Thus, it is critical to consider a new adaptive control method for further LLRERs based on the needs of patients. According to the different stages of the gait cycle, this method does not need on-demand auxiliary control methods divided into support phase and swing phase control. Also, it does not require a physical therapist to use passive and active rehabilitation training to switch between different modes. However, according to the man-machine system for rehabilitation robots, trajectory tracking and real-time error learning in patients with functional sports ability enable continuous and seamless on-demand auxiliary torque control.

**Figure 12:**
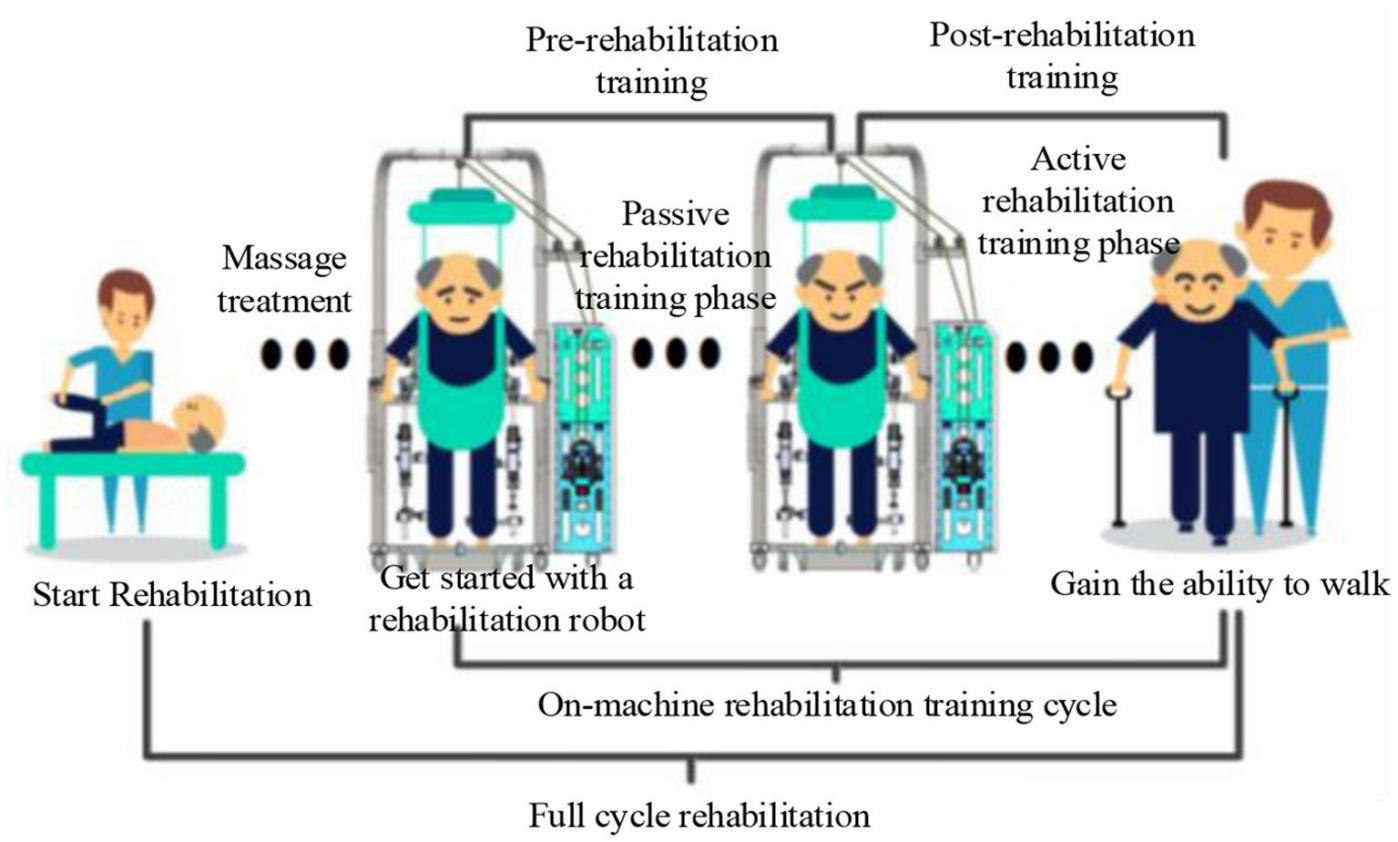
Schematic diagram of the full cycle of rehabilitation training.

According to the principles of modern hemiplegia therapy, whether a patient is different or not, they will not be in good health during rehabilitation. However, the various control algorithms ignore the improvement in the affected limb's condition. Real-time VIAC cannot be achieved by actively adjusting impedance parameters in complex situations based on the patient's health. Hence, the control strategy needs to be further improved and adjusted. In recent years, many studies have explored the LLRT approach and achieved powerful results. However, there are very few robots with full-cycle rehabilitation training (RT), which can promote positive human-machine interaction and boost user performance. In addition, existing LLRRs cannot effectively realize full-cycle gait RT. Thus, further study on HMCC based on the full-cycle RT is indispensable. In this regard, it is particularly important to develop LLRR that can adapt to various RT modes and enhance rehabilitation outcomes. Finally, Figure 12 is proposed based on the reviewed current research trends for the implementation of full-cycle rehabilitation.

The virtual reality approach will improve and play a critical role in the further growth or expansion of the lower- or upper-limb rehabilitation control aspect. In this way, the rehabilitation robot should be able to contribute to or support specific motions. The current state of the art can be categorized as “patient-cooperative” or “assist-as-needed” robots, which increase the user's power in rehabilitation by contributing only the minimal assistance required. The following benefits can be achieved during rehabilitation in a virtual reality environment with robotic control concepts. Firstly, this technology can provide direct visual feedback for the patients, making the training process more intuitive and guiding them to complete the movement tasks effectively. Secondly, virtual reality technology can achieve various game- and task-based training strategies to boost rehabilitation training. Beyond that, it can easily enhance the users' motivation to actively participate in sports-related exercise and offer a promising or optimized rehabilitation outcome (Sabah et al., 2022), as opposed to traditional therapy. However, the users are limited in the kinds of tasks they can execute in virtual environments because robots usually assist with only certain predefined tasks.

For the LLRT robot's collaborative control, two types of biomedical signals are most commonly used, including EMG and EEG. Since both signals are non-invasive, EMG and EEG acquisition methods are feasible, do not require professional medical personnel, and are safe. However, further, improvement is needed to combine them to generate a robust control signal. Normal Lokomat patient exercises are performed with a predefined gait pattern, which is achieved by position governing the linkage angle trajectories. On the contrary, it is crucial to ensure that the user is actively moving so that their lower limbs are not controlled only in a passive way through the locomotor. Thus, designing automatic gait-pattern adaptation algorithms is very significant. Furthermore, these algorithms qualify patients or users to generate some degree of voluntary locomotor ability to move or walk in the Lokomat voluntarily or actively with an unsteady or variable gait pattern. Typically, exercise with an adaptive gait pattern can promote the following benefits compared with a predefined or fixed gait pattern: active movements and muscle contractions vs. passive movements/passive muscles; more physiological and variable sensory input to the central nervous system (CNS) centers; and increased motivation of the patient, who can now control the movement of the robot. All of these benefits can truly promote a promising rehabilitation of the CNS (Jezernik et al., 2004). The predetermined haptic assistance does not allow users to attempt or intend action independently. In contrast, no assistance controller requires users to have a specific motor capability in order to drive the robot. It would be ideal for incorporating the precise trajectory haptic assistance demonstration while still allowing patients to experiment with movement on their own. The following forms of the adaptive scheme can be computed:


(1) $$\begin{array}{l} R_{k+1}=f_{R}R_{k}-g_{R}|\theta_{k}-\theta_{d,k}| \end{array}$$

Where *R*: is the control parameter that is adapted, *f*_*R*_ the robot forgetting factor; *g*_*R*_ is the learning gain; θ_*k*_ is the performance variable or measured position; θ_*d, k*_ is the desired performance variable. Designing and realizing the algorithms for automatic gait-pattern adaptation is crucial. The first algorithm generates gait-pattern adaptation by first estimating the human-robot interaction torques and then adapting the angle trajectories to encourage a reduction in the linkage or interaction torques. The second algorithm calculates the human-robot linkage torques and then converts these into the required or needed change in the trajectory accelerations. The gait-pattern adaptation can be achieved by producing a suitable variation in the reference hip and knee angle trajectories (Marchal-Crespo, 2013).

## 7. Challenges of human-robot cooperative control

A trajectory-tracking control scheme can be applied to the targeted neuromuscular. Usually, when SCI patients lack the muscle strength to move their limbs, a trajectory-tracking approach can be used to train them. However, the serious drawback of this approach is the imposition of a fixed trajectory. Thus, it may promote abnormal gait patterns and promote or encourage users who are incapable of adapting to physiological gait, making it not entirely suitable for rehabilitation robots. Furthermore, the issue of trajectory tracking control pushes the subject's limbs to follow predetermined trajectories without considering the patient's level of impairment. Precisely, the main problem with trajectory tracking and impedance control-based training is that they do not change controller parameters based on real-time initiative or the user's capabilities.

The major challenge with using hybrid techniques is the complexity of achieving the perfect amalgamation of EMG- and EEG-based techniques. However, various methods can be employed to combine bioelectricity signals; it should be noted that not all fusions are feasible or appropriate (Leerskov et al., 2020). Occasionally, the efficacy of the amalgamation method can provide inadequate outcomes as compared to the single utilization of EEG or EMG. Thus, it is crucial to consider integrating these two physiological signals within the control technique for a distinctive or specific application aimed at achieving a promising or benchmark outcome. The various combination techniques, such as the Kalman filter, simple fusion, and Bayesian fusion, can be applied for additional research. In the robotics field, the robot's ability to perform various job tasks depends on various aspects; among them, the sensor system is a very important component and one of the ways to reach expected success. Therefore, to reach the desired promising outcome, the sensor system should be programmed based on the following: (i) frequently controlling the user, robot agents, and environmental state; (ii) regularly controlling and paying close attention to the patient-robot linkage to reach an accurate interaction control system; (iii) security; (iv) defect and error tracking, (v) gaining and habitually providing sensory feedback to users for improving relearning; (vi) encouragement; and (vii) participation in the restoration training. Furthermore, sensor fusion techniques should be employed in future studies to achieve good real-time exoskeleton control or highly appropriate real-time use.

Generally, the idea of user-exoskeleton fitness and interlinkage control is vital (Rojas et al., 2021). As used for people with motor control dysfunction, the rehabilitation process includes frequent gait exercises related to the lower limb exoskeleton device (Dalla Gasperina et al., 2021). Indeed, there are enormous challenges in this regard when it comes to meeting the required kinematic compatibility of the exoskeleton structure with the users in order to provide good training. Usually, this can occur due to mismatches between wearable robot equipment and users, which can lead to joint misalignment. Thus, future research should pay close attention to overcoming this problem and providing users with comfortable, safe, and promising performance. In other words, evaluating an exoskeleton also requires compliance with wearability, which is another challenging task. Due to the complexity of lower limb movement, it's also hard to design an exoskeleton to support joint movement. Apart from that, manufacturing an exoskeleton for a relatively simple joint or coupling has become challenging due to the huge number of targets the joint involves and the added complexity imposed by the advent of spasticity. Exoskeletons that are intended to be used as support equipment for users with neuromotor adaptation do exist; however, spasticity can create a restriction that limits their applications. When there is active movement present, spasticity usually manifests. Fortunately, the electromyography (EMG) approach (Zhang et al., 2020) can be used to recognize or detect its evaluation of muscle activity. Human-robot linkage or coupling and biomechanics have mainly been carried out with rigid exoskeletons, which significantly increase the challenging inertia to the lower extremities and can result in various constraints to the user's normal kinematics (Wang and Lu, 2022). Thus, it is an important factor to consider to achieve a good coupling control system. On the other hand, there are various limitations when using EMG signals as a detection or recognition approach. This technique requires attaching electrode slices to the appropriate area of the user's skin, which is unsuitable for the user and frequently used in everyday applications. In addition, the foot pressure sensor allocation assessment cannot fit or adapt to the road surface.

The linkage force recognition of the thigh and shank requires that the exoskeleton lower limb be parallel with the user's lower limb, which is challenging or not easy to achieve due to the altered joint rotational immediate modification centers (Rastegar and Kobravi, 2021). Furthermore, the relaxation of the muscles in the lower limbs can generate a significant amount of noise or interfere with the detection or identification system. Effective dynamic modeling (Hebron and Pajor, 2021) and control are unable of adapting to changes in the payload and obstacles in the external environment. Indeed, in a number of cases, the purpose of the various control approaches for exoskeleton devices is to permit the exoskeleton to execute the appropriate fixed operation. Typically, the exoskeleton device cannot be motivated or made to adjust to some users' movements. The robot will not reach a promising outcome in this regard. For instance, an exoskeleton robot can be used in the field of rehabilitation. If the users stay passive, they tend to execute training that minimizes muscle actions and metabolism. Contrary to this, the user impedes or resists exoskeleton motion when patients are permitted to move actively. Thus, this will yield uneven muscle activity, which can limit reaching the required training outcome. Hence, robust algorithms are needed to overcome these issues, which can promote the promising user-device cooperative control required (Zhong et al., 2021). However, there are still many issues that need to be resolved, such as the difficulty of precise modeling and the absence of stability proof.

## 8. Conclusions

Lower limb exoskeletons play a significant role in various applications, including rehabilitation, and as medical assistants. They sometimes can help healthy people execute different tasks (for instance, military and industrial workers). The rehabilitation area (medical field) can make extensive use of power assistance for elderly or frail people to help patients resume a normal life and enhance their quality of life. In this examination, the background and development of exoskeletons, actuation control approaches, classifications, and their various applications have been discussed. Thus, two popular control approaches, namely the physiological-based control (sEMG and EEG) and the traditional-based cooperative control for the lower limb exoskeleton have been reviewed, and an in-depth comparison has been presented. Moreover, the benefits and drawbacks of human movement intention detection approaches that rely on physiological signals and human-robot interaction data have been highlighted. We discussed the trends of cooperative control (multiple information fusion) since the combination of two bioelectrical signals can significantly increase recognition accuracy. Finally, based on the reviewed articles, it can be concluded that the virtual reality approach can improve and play a significant role in developing the lower or upper limb rehabilitation control aspect. Thus, future rehabilitation can explore virtual reality as a significant part of a larger rehabilitation setup that provides rich multimodal stimuli and direct dynamic interaction between the user and a smart machine.

## Author contributions

GM wrote the first draft of the manuscript. XZ contributed to conception and supervision of the study. RD, AA, KH, and EM organized the graphs and tables. All authors contributed to manuscript revision, read, and accepted the submitted version.
